# Voluntary action as problem-solving: an functional magnetic resonance imaging study

**DOI:** 10.1093/cercor/bhaf318

**Published:** 2025-11-24

**Authors:** Silvia Seghezzi, Daniel Yon, Patrick Haggard

**Affiliations:** Institute of Cognitive Neuroscience, University College London, Alexandra House, 17-19 Queen Square, London WC1 3AZ, United Kingdom; Birkbeck, University of London, Malet St, London WC1E 7HX, United Kingdom; Birkbeck, University of London, Malet St, London WC1E 7HX, United Kingdom; Institute of Cognitive Neuroscience, University College London, Alexandra House, 17-19 Queen Square, London WC1 3AZ, United Kingdom

**Keywords:** action, planning, self-generated, supplementary motor area, voluntary behavior

## Abstract

The capacity to pursue goals, across a series of intermediate stages, is a distinctive achievement of human cognition. Scientific investigations of goal-directed action have emphasized either of two different aspects of this capacity. Research on executive function has described coordination of extended action sequences that solve multi-part problems. Meanwhile, research on voluntary action has emphasized the processes of endogenous generation and autonomy, which are essential for many complex problems, particularly those involving creativity and insight. Because many complex problems can be solved in several ways, choosing and generating a path through the problem space requires a convergence of executive intelligence and volitional control. Here we use fMRI to explore the links between volition and problem-solving in the human brain. Participants performed the Tower of London task (a classical neuropsychological problem-solving challenge) either by generating their own solutions or by following stepwise instructions for each move. We showed behavioral signatures of action planning, associated with a distributed network of frontal and parietal activations, when participants generated their own solutions. We also showed the crucial role of medial frontal cortex, traditionally associated with endogenous generation of very simple willed actions, in goal-directed problem-solving, based on its connectivity with a wider prefrontal network.

## Introduction

Goal-directed action is a central feature of human cognition and behavior, and is observed—though to a lesser extent—in other animals (Clayton et al. 2003). The capacity to pursue one’s own projects and goals extended in space and time has historically been identified in philosophy and political theory with the good life (Hegel 1820; Marx 1844; Sen 1979). This capacity has been studied in cognitive neuroscience and psychology under various headings, notably executive function and action planning.

A rich tradition of work in executive function has investigated the mental processes and brain mechanisms that allow us to coordinate extended sequences of actions to solve a problem or reach a goal (Gilbert and Burgess 2008). This work has identified three key constructs—sequencing, hierarchy, and optimisation—that support goal-directed behavior. Sequencing refers to the combination of several primitives in particular ordered patterns that should eventually achieve the goal (Koechlin et al. 2000; Koechlin and Summerfield 2007). Hierarchy refers to the capacity to represent and activate subsequences of several action elements as a single chunk, enabling the efficient composition of more complex action sequences from several simpler ones (Badre 2008; Botvinick 2008). Optimisation refers to the process of selecting, out of the multiple or even infinite ways of solving a problem, the one that is best according to some current policy (Cooper and Shallice 2000; Cisek 2007; Cisek and Kalaska 2010; Shenhav et al. 2013).

However, for many complex problems there may be several paths that are all equally optimal. Even relatively mundane goals, like walking through a familiar city (Linkenauger et al. 2019) or shopping in a supermarket without doubling back on ourselves (Shallice and Burgess 1991) can be achieved in multiple different ways. In these situations, the ability to select between alternatives, and to generate the selected action, remains present, yet must clearly rely on something other than optimisation. Some endogenous signal must break the symmetry between two equivalent options, and generate an action, in order to advance the path towards the goal (Gazzaniga 2012). This generative capacity is often linked to self-generated, volitional actions mediated by the voluntary motor system (Haggard 2019). Despite these clear interdependencies, research on volition and executive function has proceeded largely independently, with separate experimental traditions and theoretical frameworks (Shallice and Burgess 1991; Haggard 2008).

The concept of “self-generated action” is central to the cognitive neuroscience of voluntary action. Classically, it has been operationalized by contrasting actions generated by the participant’s own choices over whether, when and how to act with actions performed in response to an external imperative stimulus (Brass and Haggard 2008; Zapparoli et al. 2017). This contrast links self-generated action to the medial frontal cortices, notably the pre-supplementary motor area (pre-SMA) (Fried et al. 1991; Lau et al. 2004; Passingham et al. 2010; Seghezzi et al. 2019), though wider prefrontal (Frith et al. 1991) and parietal networks also contribute (Desmurget and Sirigu 2009). However, this literature has been criticized for relying on unecological contexts, in which action choices have no real significance (Maoz et al. 2019; Mudrik et al. 2020). Further, pre-SMA activations might reflect generic processes such as timing decision, rather than generation of volitional action per se. Overviews of the neuroscience of human volition might make one wonder why volition is considered central to human nature, given the impoverished and unnatural scenarios that neuroscientists have used to study it (Dennett 1984).

Several researchers have accordingly tried to enrich the notion of voluntary action that emerges from laboratory studies. One approach involves giving actions meaningful consequences (Maoz et al. 2019). This raises the possibility that the action is no longer truly endogenous: the representation of the outcome might effectively become an imperative stimulus (James 1890).

Here, we explore a very different approach, by embedding individual voluntary actions within a rich chain of goal-directed problem-solving. As an agent generates a series of action steps progressing towards a final goal, they freely choose at each step which of several actions to make. Agents are typically assumed to aim at “good” solutions (i.e. those that take an optimal path through the problem-space). Nevertheless, agents readily solve problems involving choices between actions that are strictly equivalent. This fact demonstrates that self-generated action is then essential to problem-solving.

We asked participants to perform several variants of the neuropsychological Tower of London (ToL) task (Shallice and Burgess 1991). This task involves a series of action steps, moving three colored balls between three pegs in order to reproduce a goal configuration of the “tower.” Thus, participants are required to plan and then execute their own sequence of actions to reach the “goal” state (self-generated condition). We developed a novel stimulus-driven control condition in which an imperative stimulus told participants at each step which ball to move to which peg, without any representation of an overall goal, nor any requirement to choose or plan any move. The contrast between self-generated and stimulus-driven actions has classically been used to identify the processes underlying voluntary action (Passingham 1987). Here we recapitulate this classical contrast in the context of complex, “intelligent” behaviors having a clear link to cognitive theories of intelligence (Shallice 1982).

The latency of each move was analyzed to provide behavioral measures of planning and choice processes. Functional magnetic resonance imaging (fMRI) was used to identify brain activations characteristic of self-generated action solutions. Our analyses identified a network of frontal and parietal brain regions, including the pre-SMA, that strongly overlapped with previous studies contrasting simple voluntary with simple stimulus-driven actions. Additional analyses of functional connectivity from the pre-SMA to the whole brain showed that the pre-SMA is connected to other frontal and prefrontal regions prior to the first move of the sequence in the self-generated condition. In contrast, during the planning and execution of subsequent moves, the pre-SMA shows increased interaction with the parietal cortex, likely reflecting a role in monitoring ongoing performance and guiding action execution.

In sum, these results confirm that a brain area classically associated with simple volitional actions plays a key role in the more meaningful, enriched contexts of goal-directed problem-solving. Most previous neuroscientific studies of voluntary action have focused on the capacity for endogenous generation of movement. Our results suggest that volition should be seen within a wider cognitive context, where endogenous action generation enables intelligent problem-solving.

## Methods

### Participants

Aligning with previous fMRI studies involving the Tower of London task, 26 healthy, right-handed participants (8 males, mean age 22 years, SD 4.4, range 19–33 years) participated in the study. None had a history of neurological or psychiatric disorders. Color-blind individuals were also excluded as the tasks crucially required detecting color differences. All inclusion and exclusion criteria were established before data collection commenced. All participants gave informed written consent. The study was approved by University College London Research Ethics Committee (approval code: 1825/003).

### Experimental task

**Fig. 1:**
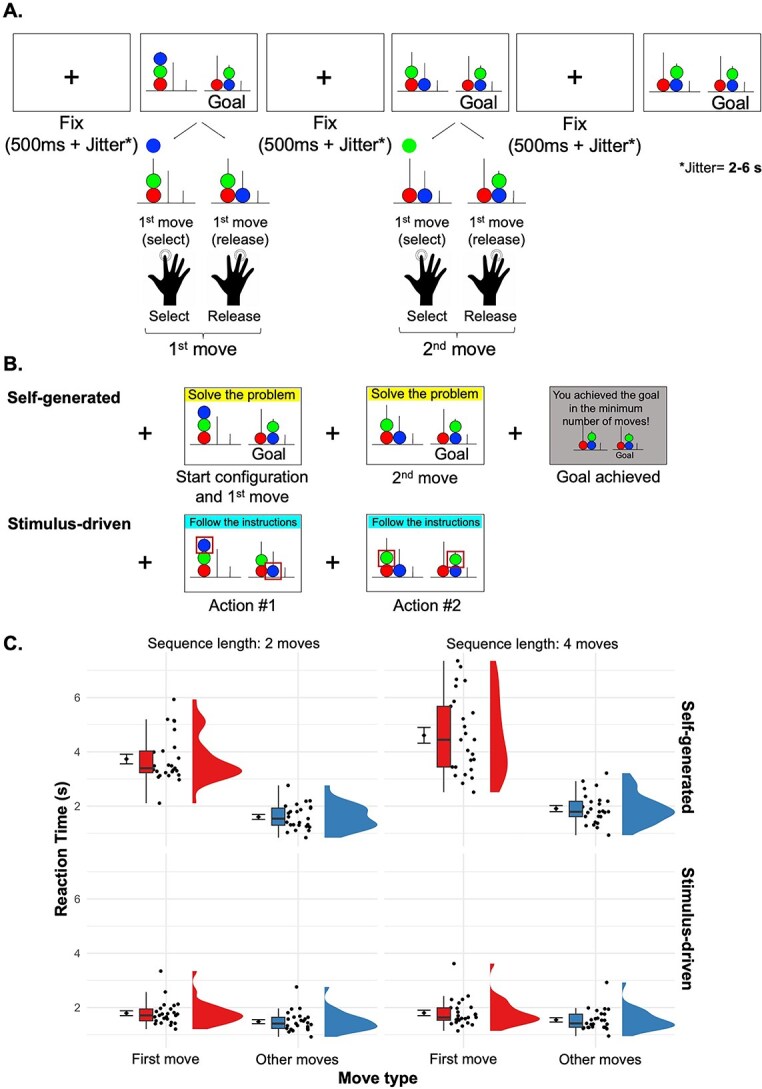
Experimental manipulations of self-generated action in the Tower of London task: (a) Schematic of the experimental task, based on a modified version of the Tower of London paradigm. Participants solved problems by moving colored balls between pegs one at a time, to match a goal configuration in the minimum number of moves as possible. (b) Experimental conditions. In the self-generated condition, participants saw the goal configuration from the start of the trial, so could choose and plan each action in advance. In the stimulus-driven condition, a red rectangle indicated at each step first which ball to select and then where to place it, guiding each move. (c) Behavioral results. Raincloud plots show the distribution of mean reaction times (RTs, in milliseconds) across 26 participants. Each dot represents an individual participant; boxplots display the group-level median and range. The larger central dot indicates the group mean with associated standard error. Although raw RTs are plotted here for interpretability, statistical analyses were performed on log-transformed RT values (Osborne 2002). First-move RTs were substantially longer than subsequent moves in the self-generated condition only, and especially for longer sequences. This is consistent with a planning process linked to self-generated action in problem-solving contexts.

We developed a modified, computerized version of the Tower of London task, a well-established paradigm frequently used to assess problem-solving abilities in healthy participants and neurological patients (Shallice and Burgess 1991). The task presented participants with a series of 2D arrangements of colored balls positioned on three pegs of different lengths. The aim was to transform the initial arrangement into a goal configuration by using the minimum number of moves, all while adhering to specific rules. Only one ball could be moved at a time. Each ball could be moved from one peg to another, with the constraint that no more than three, two, and one ball could be placed on the first, second, and third peg, respectively. If more than one ball was located in one peg, only the ball occupying the highest position could be selected (in line with the classical, physical version of the task) Fig.  1a.

In our modified task, the goal was presented on the right side of the screen and remained available until the goal was achieved. The initial arrangement was presented on the left side of the screen and was progressively updated by participants’ actions on the tower configuration.

Each move within the tower configuration required the participant to perform two distinct button presses. The first button press selected the specific ball to be moved, determining the ball to be lifted from the peg. The second button press indicated the peg where the ball needed to be placed, determining the release of the selected ball on it.

Participants solved the problems using the index, middle and ring fingers, with each finger corresponding to the selection of a specific peg. Upon reaching the goal, participants were presented with a screen reporting the goal configuration on a gray background, indicating whether they successfully accomplished the problem within the minimal number of moves or not.

A time interval of 250 ms plus a jitter interval ranging from 2.5 to 6.5 s was introduced between subsequent moves. During this time interval, a fixation cross was presented on the screen. The experiment was run using Psychtoolbox v.3.0 (Kleiner et al. 2007).

### Problem selection and conditions

A total of 72 Tower of London problems were used, drawn from 12 distinct “families” expressed in six different color permutations. Half of the problems required two moves to be solved, while half required four optimal moves. The selection of problems was carried out using the Tower Tool software tool developed specifically for analyzing tower tasks (Kaller et al. 2011). Notably, all chosen problems had a singular optimal solution and were devoid of any detours or dead-ends.

The experimental consisted of four 10 min blocks. Within each block, problems were randomly selected while ensuring that problems from the same family were not included in the same run.

Within each block, problems were randomly selected. Half of problems required self-generated solutions, where they were responsible for planning and executing a sequence of problem-solving actions based on the goal provided for them (Fig.  1b, yellow). In a stimulus-driven control condition, they made the same moves without seeing the goal. Instead, they followed a series of instructions about which action to take next (Fig.  1b, light blue), implementing a pre-specified solution. We measured the reaction time to pick up (and then move) each ball as a behavioral measure of the cognitive processing in each condition.

Whether participants were in the self-generated or other-generated condition was indicated through a colored band positioned on the top of the screen—displaying the instruction “solve the problem” or “follow the instructions” (see Fig.  1b).

Participants solved as many problems as possible in 10 min, earning an additional bonus of 0.5 pence for each problem completed in the minimum number of moves. This incentivized both accuracy and a reasonably short solution time for each problem.

### Procedure

The experiment started with obtaining participants’ informed consent, which was followed by a preliminary introduction and training phase before the actual scanning procedure. The training was structured into three phases. After reading the instructions, participants were asked to solve two problems, one requiring two moves and another involving four moves, under the intention-driven condition. Participants were then instructed to the stimulus-driven condition and asked to solve two additional problems, again consisting of two and four moves, but under the stimulus-driven condition. Subsequently, participants solved a mixed set of 12 problems including both intention-driven and stimulus-driven scenarios, presented in a randomized order. During the training phase, participants solved the problems using the laptop keys J, K, and L. However, in the actual scanner experiment, a response box was provided, with each button positioned beneath the index, middle, and ring fingers.

After completing the training phases, participants underwent an MRI safety interview with the radiographer to ensure their MRI compatibility. Following this, participants were positioned within the fMRI scanner. An initial practice session was conducted inside the scanner to help participants become accustomed to the response box. This practice involved solving two problems, one for each experimental condition.

After the experiment, participants were provided with a debriefing that explained the primary aims of the study, and were compensation at a rate of £9.20 per hour.

### MR image acquisition and preprocessing

Scanning took place at the Wellcome Centre for Human Neuroimaging, University College London, using a 3 Tesla Siemens Prisma MRI scanner with a 64-channel head coil (Siemens Healthcare, Erlangen, Germany).

Functional T2*-weighted images were acquired over four sessions each lasting ~ 15 min. The sequence was optimized to minimize signal dropout in the orbitofrontal cortex using a slice tilt of −30° and a z-shim of −1.4. The volume TR was 3.36 s, with a TE of 30 ms and echo spacing of 0.5 ms. Per volume, 48 slices were collected in transverse orientation, resulting in a matrix size of 64 × 72 and a 3 mm isotropic voxel size. After two functional sessions, we also acquired a fieldmap with the following parameters: short TE = 10 ms, long TE = 12.46 ms, polarity of phase-encode blips = −1, applied Jacobian modulation = no, total EPI readout time = 36 ms, in an ascending slice order. The structural images were collected using a T1-weighted sequence with 1 mm isotropic resolution.

All MRI pre-processing was performed using SPM12 (Statistical Parametric Mapping 12; Wellcome Centre for Human Neuroimaging, London, UK). The anatomical images were segmented into gray matter, white matter, and CSF maps and normalized to the Montreal Neurological Institute (MNI) template. The first five functional images were discarded to allow for signal equilibrium. Functional data were then realigned and unwarped (including distortion correction with fieldmaps) and coregistered to the anatomical image. Forward deformation fields from the anatomical image were then used to normalize the functional images into MNI space. Finally, functional images were smoothed with an 8 × 8 × 8 mm kernel FWHM.

### Behavioral data analysis

All analyses were performed by means of the statistical software R (4.0.3) and the lme4 package (Bates et al. 2014). We estimated the effects of move (First vs. Other), condition (Self-generated vs. Instructed) and sequence length (2 vs. 4 moves) and their interactions on Reaction Times (RTs). RTs were log-transformed to better approximate the normal distribution than raw RTs. An initial model was estimated the maximal random structure [41] corresponding to by-participant intercept and by-participant random slopes for each effect included as a fixed effect. Since the maximal model did not reach convergence, the random structure was simplified by iteratively removing one term (starting from interactions, then moving to simple effects). Since convergence was reached in three models with two random slopes (see Supplementary Material), these models were compared based on the Akaike Information Criterion (AIC), and the results of the model with the smallest AIC were explored. Post-hoc planned comparisons were run with the package emmeans and p values were corrected using the Bonferroni method.

The data and the R script for behavioral analysis are available at the following link: https://osf.io/ecsqa/?view_only=c4a1f68c35ae4c2a8d2e991f65e67f5d.

### Univariate neuroimaging analysis

We constructed a general linear model with event-related regressors aligned to onset of ToL screen (i.e. graphical depiction of the current state and of the goal state) for each move. The duration of each regressor was coded as the time taken from stimulus onset to the second button press (i.e. release of the moved ball into the desired peg). A different regressor was used for each combination of the factors move (First vs. Other), condition (Self-generated vs. Instructed) and sequence length (2 vs. 4 moves). Each regressor was also associated with one parametric modulator, corresponding to the number of button presses for that move. This was done to account for the possibility that -although the expected number of button presses per event was two (one for selecting which ball to move and one to release it)- participants could potentially make mistakes by selecting an empty peg or by trying to release a ball in a peg with no empty spaces. Regressors corresponding to the fixation cross during the inter-move interval were included as nuisance regressors, as were movement parameters. Regressors were specified per scanning run. For each combination of the factors “move” and “sequence length,” first-level the t-contrast “Self-generated > Instructed” was computed. A full-factorial design containing the variables “move” and “sequence length” was adopted for second-level analyses.

Correction for multiple comparisons was applied at the cluster-level (*P* < 0.05, family-wise error corrected), using a cluster-forming threshold of *P* < 0.001, uncorrected.

### Psychophysiological interaction analysis

To assess changes in connectivity between the pre-SMA and whole brain activity, we carried out a generalized psychophysiological interaction (gPPI) analysis. gPPI is a measure of context-dependent connectivity, explaining the regional activity of other brain regions (here, whole brain) in terms of the interaction between responses in a seed region and a cognitive or sensory process. Here, as a seed region we used the pre-SMA as defined by the SPM anatomy toolbox 3.0.

We carried out gPPI analysis using the toolbox Functional Connectivity (CONN) toolbox [42]. The pre-processing of resting-state fMRI data was conducted according to the default pipeline included in CONN-fMRI Functional Connectivity toolbox (version 187) including realignment and unwarping, slice-timing correction, structural segmentation and normalization, functional normalization, outlier detection (Artifact Detection Tool, conservative settings: 95 percentiles in normative sample), smoothing (10-mm Gaussian kernel) and band-pass filtering (0.008 < f < inf Hz) to reduce the effect of low-frequency drift and high-frequency noise. The CONN toolbox then used a component-based noise correction method (CompCor) to identify and remove the principal components of physiological and other sources of noises from white matter and cerebral spinal fluid. Additionally, the confounding effect of the movement-related parameters (six dimensions with their first order derivative) was removed.

As a first level, we specified four different conditions resulting by the combination of the factors move (First vs. Other) and sequence length (2 vs. 4 moves). The analysis was performed on the self-generated condition only. Realignment paraments were added as covariates.

The second level was performed in SPM. A full-factorial design containing the variables “move” and “sequence length” was adopted for second-level analyses. Correction for multiple comparisons was applied at the cluster-level (*P* < 0.05, family-wise error corrected), using a cluster-forming threshold of *P* < 0.001, uncorrected.

## Results

### Manipulating volition in the Tower of London task

We asked participants (*n* = 26) to complete a variant of the Tower of London problem-solving task while we recorded their brain activity using fMRI.

Participants had higher reaction times for the first move of a new problem compared to the other moves, in self-generated but not in stimulus-driven conditions (Effect Condition × Move Type (first vs. other): F(1,7328.9) = 93.69, *P* < 0.001)). This effect was more pronounced on problems requiring more moves to reach the goal (Interaction effect of Condition × Move Type (first vs. other) × Sequence length (2 vs. 4 moves): F((17328.5) = 5.98, *P* = 0.014), Fig.  1c). This pattern of results is consistent with a time-consuming process of planning the series of steps required to reach the goal in the self-generated condition only, with this process preceding at least in part the first move.

### Neural correlates of self-generated action solutions overlap with “simple” volition networks

**Fig. 2:**
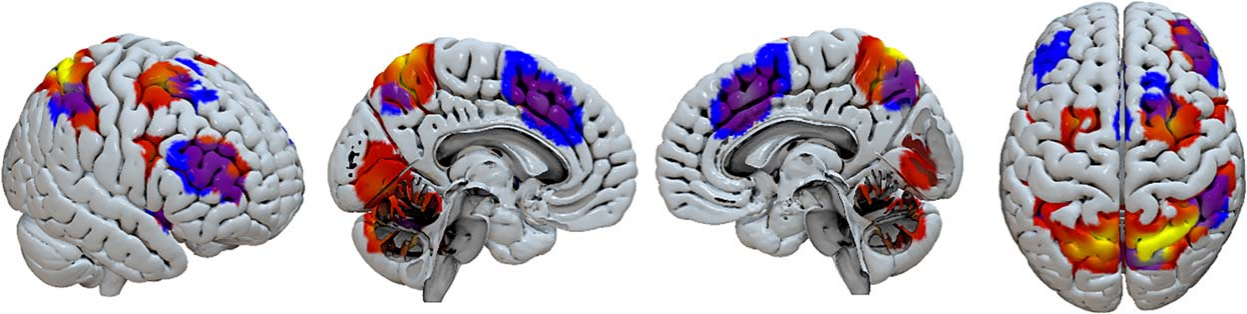
Overlap between brain activity observed in our Tower of London task (contrast: self-generated > stimulus-driven; shown in red/yellow) and analogous contrasts derived from a meta-analysis of 24 neuroimaging studies of volition (shown in blue). Regions of overlap appear in purple.

To reveal the neural mechanisms involved in the self-generation of these problem solutions, we identified regions across the whole brain showing greater activity during self-generated compared to stimulus-driven trials. This revealed a range of frontal and parietal brain areas—including regions of the medial frontal cortex, notably the pre-SMA (Fig.  2, in red/yellow).

### Overlap between “complex” and “simple” volition brain networks

To assess the overlap between the network we identified here and previous sensorimotor studies of “simple” volition, we conducted neuroimaging meta-analyses of previous studies contrasting self-generated with stimulus-driven actions (see Supplementary Material, Supplementary Tables S12 and S13). The regions associated with self-generation in our problem-solving context consistently overlapped with those identified in simpler, sensorimotor volition tasks (Fig.  2, in blue).

**Fig. 3:**
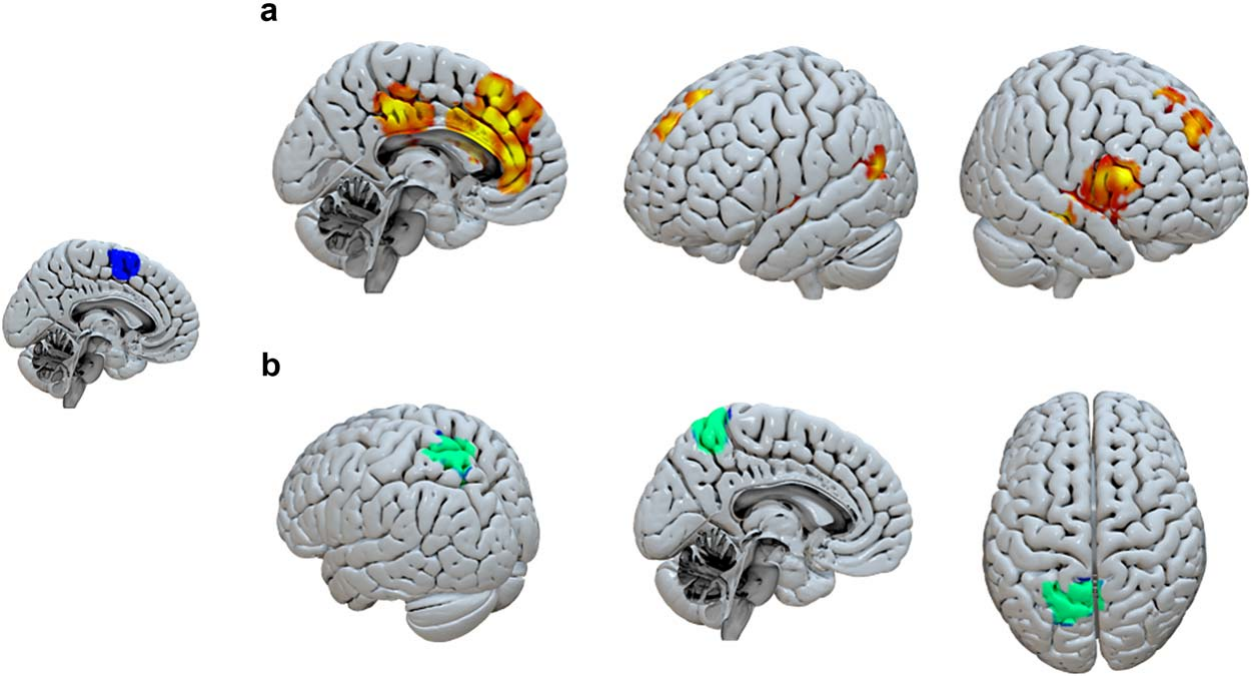
Functional connectivity results from the gPPI analysis using the pre-SMA as a seed region (seed shown in blue, left panel). (a) During the initiation of internally generated solutions, the pre-SMA showed increased connectivity with prefrontal regions, including the IFG and cingulate cortex (shown in red/yellow). (b) As participants proceeded to execute subsequent moves in the sequence, the pre-SMA functional connectivity shifted towards connection with parietal regions, particularly the superior parietal lobule (shown in green/blue).

We next investigated how the pre-SMA contributes to complex problem-solving by using whole-brain connectivity analyses (gPPI) to estimate the changing pattern of pre-SMAs co-activation with other regions at different stages of the task. We found the pre-SMA was functionally connected to prefrontal regions like Inferior Frontal gyrus (IFG) and cingulate cortex before the first move of the sequence (see Fig.  3a), but it became functionally connected to posterior brain regions, notably the Superior Parietal lobule (see Fig.  3b), as subsequent moves of the sequence unfolded. This suggests that the pre-SMA plays an important role in coordinating first the planning and then the implementation of complex self-generated action solutions, through links to other cortical association areas.

## Discussion

The capacity to freely generate one’s own course of action is central to the ability to solve complex problems in the real world, as well as to innovation, discovery and societal progress. Interestingly, this distinctive feature of human mental life has tended to fall between two traditions of cognitive neuroscience research. Executive function studies have long investigated problem-solving (Norman and Shallice 1986; Goel and Grafman 1995; Cooper and Shallice 2000; Daw et al. 2005; Wagner et al. 2006; Mushiake et al. 2009; Cisek and Kalaska 2010; Gonen-Yaacovi et al. 2013). These studies have the more or less explicit assumption that the processes of action generation are entirely downstream from the “intelligent” processes that find solutions to problems. Conversely, laboratory studies of volition focused on very simple voluntary actions, and therefore struggled to express why voluntary control plays the important role that it does in individual and collective human life (Haggard 2019). Agents serially and routinely select successive voluntary actions to chart multiple paths through complex problem spaces and achieve their goals (Coutrot et al. 2018). Recent and timely focus on enriching the neuroscience of voluntary action has often focused on value rather than complexity (Maoz et al. 2019), while recent research on complexity of human action sequences has generally favored instructed rather than self-generated actions (Koechlin and Hyafil 2007). Therefore, the conceptual gap between volition and intelligence persists.

Our study represents a first step towards filling this gap. First, we confirm that complex problem-solving scenarios rely on the same brain mechanisms as simpler voluntary actions. Importantly, this includes the putative “volition centre” in the pre-SMA, historically identified with endogenous, voluntary action (Lau et al. 2004; Passingham et al. 2010) and even sometimes with “free will” (Hallett 2007). Interestingly, some authors have critiqued the association between pre-SMA and volition, suggesting instead that this area encodes or supports features that are merely incidental to self-generated movement, such as the timing of an unconstrained key press (Jahanshahi et al. 1995; Jenkins et al. 2000; Nachev et al. 2008) but that are not constitutive of volition. These critiques may seem reasonable, given that the core features of volition are elusive (Haggard 2019). Nevertheless, our results suggest that the pre-SMA activation is genuinely related to the self-generated quality of some actions. Specifically, solving the Tower of London requires agents to choose which of several actions to make. It removes the problematic instruction to “act whenever you feel like it” that pervades the voluntary action literature (Libet et al. 1983). Our results showed clear pre-SMA activation in the context of planning meaningful self-generated actions that solve problems. This context avoids many of the incidental, epiphenomenal elements that make classical voluntary action studies controversial and difficult to interpret (Dominik et al. 2024).

The extended context that complex problem-solving provides for volitional action also allowed us to identify an extended brain network in which an enriched concept of volition plays an important role. A surprising feature of many (but not all, Soon et al. 2008) neuroimaging studies of volitional action is the absence of volition-related activations in any areas upstream of pre-SMA (Brass et al. 2013). In particular, neuroimaging studies of voluntary action generally do not report wide prefrontal, frontopolar or even lateral frontal activations, even though large neuroimaging studies integrating several executive function tasks consistently show activations in a distinct set of prefrontal territories forming part of a so-called Multiple Demand System (Duncan et al. 2020; Chiou et al. 2024; Shashidhara et al. 2024). Barring metaphysical oddities such as uncaused causes or mind–body causation, one might conclude either that pre-SMA neurons themselves have some endogenous generative or regenerative capacity, like other central and autonomic generators (Marder and Bucher 2001), or else that pre-SMA neurons responsible for volition receive driving input from areas not normally considered in studies of volition, such as the ascending subcortical input from the basal ganglia (Jahanshahi et al. 2015).

Thus, it remained unclear how thoughts, in a very general sense, could interface to action representations, in the Pre-SMA. Our study contributes to solving this puzzle. Using gPPI functional connectivity analysis we showed that the Pre-SMA is not, in fact, self-starting or self-sufficient in voluntary action. Rather, the Pre-SMA has connections with specific medial and lateral prefrontal areas. One of these, the anterior ACC, is thought to evaluate the motivational significance of potential options and resolve conflict between competing goals (Holroyd and Yeung 2012; Shenhav et al. 2013). This area might therefore establish when an internally generated action should be initiated. Another of these connected areas, the right inferior frontal gyrus pars triangularis, is often discussed in the context of language. This area’s role in syntactic structure appears to reflect a more general capacity for combinatorial and hierarchical processing (Koechlin et al. 2003; Badre 2008)—one that may also support the assembly of complex action plans in problem-solving contexts.

Prefrontal regions including the IFG are particularly activated when agents begin to plan a strategic solution to a complex problem (Koechlin et al. 2003). Our gPPI analysis suggests that this activation is functionally connected to the activity in the pre-SMA when these solutions are being freely generated by the agent themself. This suggests that activity in “volition centres” like the pre-SMA is not an epiphenomenon of the rather unnatural tasks used to elicit volition in laboratory settings, but instead reflects the important contribution that volitional processing can make to solving problems and achieving goals in rich environments and over more extended timescales (Passingham et al. 2010).

Our study also speaks to the interesting and controversial question of the respective roles of frontal and parietal networks in volition. The involvement of prefrontal cortex in the voluntary control of behavior has a long history, beginning with Phineas Gage (Damasio et al. 1994). However, several EEG (Sirigu et al. 2004) and brain stimulation (Desmurget et al. 2009) studies point to a contribution of parietal cortex in the subjective experience of volition. Our gPPI analyses clearly show that pre-SMA is functionally connected first with the prefrontal cortex during the planning phase of goal-directed complex action sequences, and subsequently, during the execution of later actions within the sequence, with parietal areas such as the superior parietal lobule. These results confirm that both frontal and parietal nodes both play key roles in complex volitional behavior. However, these roles appear quite different. We hypothesize that prefrontal cortex plays a role in planning and initiation of volitional problem-solving actions, while parietal cortex would, in contrast, monitor the current state of progress of an action sequence, perhaps with a view to chaining the next movement element (Fontana et al. 2012).

Our results can also usefully be interpreted within the framework of dual-loop and meta-loop models of cortical organization (Weiller et al. 2022; Weiller et al. 2025). According to this view, the key difference between our self-generated and instructed conditions might lie in how the various movement steps are organized to achieve a distal goal. In our control condition, participants executed a sequence of moves step by step, following an instructed stimulus. This process is consistent with dorsal pathway involvement in externally-guided sensorimotor transformations (Rauschecker and Scott 2009). In contrast, the experimental Tower of London condition required participants to generate a hierarchical structure of steps, including mental simulation of intermediate moves, and comparison of alternative strategies. These operations are more closely associated with ventral pathway functions (Cisek and Kalaska 2010; Kilner 2011). The dorsal and ventral streams eventually converge in lateral hubs such as the IFG and inferior parietal cortex (IPC), which are proposed to support hierarchical concatenation and “syntactic” organization across cognitive domains (Weiller et al. 2022). At the same time, medial hubs including the pre-SMA, anterior cingulate cortex, and precuneus contribute to the action evaluation and selection and the initiation of internally generated actions (Passingham 2021). Together, these lateral and medial hubs are argued to form a “meta-loop” linking internal cognition to external contexts for action through a “multiple-demand system” (Duncan et al. 2020). Thus, while the conditions of our experimental design aimed to separate internally-generated from externally-triggered routes to action, in the world outside the laboratory, these two systems must collaborate through a “meta-loop” to achieve most everyday cognitive tasks. From this perspective, the pre-SMA should not be seen in isolation, but rather as a node within a distributed architecture that integrates perceptual, mnemonic, and executive resources to generate complex, goal-directed behavior. Classical views of pre-SMA as “the locus” of internally-generated action (Goldberg 1985; Passingham et al. 2010) could be updated to take into account this network-based view, informed by our task-related gPPI analysis findings.

In sum, our findings reveal a striking overlap between the brain networks for volitional action and those engaged during complex, goal-directed thought and problem-solving. By embedding self-generated and stimulus-driven conditions within an extended, goal-directed problem-solving context, we show that the pre-SMA and its wider frontal–parietal connected network play a key role not only in simple endogenous movement initiation but also in the flexible generation of action sequences required to reach future goals. The capacity for voluntary action is enlisted to solve current challenges, by interfacing the executive areas that underlie cognitive control to the cortical motor networks for endogenous action. Thus, volition is not confined to isolated random motor acts but is deeply embedded in higher cognition. Thought and action are more integrated than one might surmise from previous neuroimaging literature.

## Supplementary Material

SupplementaryMaterials_bhaf318
