# Supplementary material for: Voluntary action as problem-solving: an functional magnetic resonance imaging study
Source: Cereb Cortex. 2025 Nov 24;35(11):bhaf318. doi: 10.1093/cercor/bhaf318 (PMC12642695; doi:10.1093/cercor/bhaf318)
Supplement: SupplementaryMaterials_bhaf318 [file supplementarymaterials_bhaf318.docx]

***Voluntary action as problem-solving: an fMRI study***

Running title: Voluntary action as problem-solving

Silvia Seghezzi^1,2^, Daniel Yon^2^, & Patrick Haggard^1*^

1 Institute of Cognitive Neuroscience, University College London, London WC1 3AZ, UK

^2^ Birkbeck, University of London, London WC1E 7HX

Correspondence: Patrick Haggard, Institute of Cognitive Neuroscience, University College London, London WC1 3AZ, UK [p.haggard@ucl.ac.uk](mailto:p.haggard@ucl.ac.uk)

**Supplementary material**

Behavioural Results

The best model according to the AIC criterion (See Table S1) was explored, revealing a significant effect of move type (F(1,196.0)= 183.37, p< 0.001), indicating longer RTs for the first move (M= 2.98; SD= 1.52) than other moves in the sequence (M= 1.63; SD= 0.47). The effect of condition was also significant (F(1,113.6)= 30.62, p< 0.001), indicating longer RTs for the self-generated (M= 2.96; SD= 1.55) than instructed condition (M= 1.65; SD= 0.45). The effect of sequence length was also significant (F(1,7328.8)= 78.24, p< 0.001) indicating longer RTs for 4 moves problems (M= 2.47; SD= 1.50) than 2 moves problems (M= 2.15; SD= 1.08).

The interaction between move type and condition was significant (F(1,7328.9)= 93.69, p< 0.001), indicating a greater difference between first move and other moves in the self-generated condition (first move M= 4.17; SD= 1.26; other moves M= 1.76; SD= 0.53) than in the instructed condition (first move M= 1.80; SD= 0.47; other moves M= 1.51; SD= 0.38). The interaction between condition and sequence length was also significant (F(1,7328.4)= 36.64, p< 0.001) indicating a greater difference between 4 moves and 2 moves in the self-generated condition (2 moves M= 2.66; SD= 1.28; 4 moves M= 3.26; SD= 1.74) than in the instructed condition (2 moves M= 1.63; SD= 0.43; 4 moves M= 1.67; SD= 1.74).

The three-way interaction between move type, condition and sequence length was significant (F(1,7328.5)= 5.98, p= 0.014). In particular, the difference between first move and other move seems to be greater for 4 moves problems than 2 moves problems in the self-generated condition (first move 2 moves M= 3.73; SD= 0.88; other moves 2 moves M= 1.60; SD= 0.45 vs. first move 4 moves M= 4.60; SD= 1.44; other moves 4 moves M= 1.90; SD= 0.56, t(7330)= 2.34, p= 0.038 Bonferroni-corrected) than in the instructed condition (first move 2 moves M= 1.7; SD= 0.44; other moves 2 moves M= 1.45; SD= 0.37 vs. first move 4 moves M= 1.80; SD= 0.50; other moves 4 moves M= 1.55; SD= 0.39, t(7328)= -1.19, p= 0.526 Bonferroni-corrected). See **Table S1**.

**Table S1. Results of the model comparison procedure for Reaction Times (RTs).**

| **Dependent variable** | **Model** | **Convergence** | **Parameters** | **AIC** | **logLikelihood** | **Deviance** |
| --- | --- | --- | --- | --- | --- | --- |
| Log(RTs) | 1+(1+Move*Condition*SequenceLenght\|ID)+Move*Condition*SequenceLenght | Model failed to converge | -- | -- | -- | -- |
|  | 1+(1+Move*Condition+SequenceLenght\|ID)+Move*Condition*SequenceLenght | Model failed to converge | -- | -- | -- | -- |
|  | 1+(1+Move+Condition*SequenceLenght\|ID)+Move*Condition*SequenceLenght | Model failed to converge | -- | -- | -- | -- |
|  | 1+(1+Move*SequenceLenght+Condition \|ID)+Move*Condition*SequenceLenght | Model failed to converge | -- | -- | -- | -- |
|  | 1+(1+Move+Condition+SequenceLenght\|ID)+Move*Condition*SequenceLenght | Model failed to converge | -- | -- | -- | -- |
|  | 1+(1+Condition+SequenceLenght\|ID)+Move*Condition*SequenceLenght | Yes | 15 | 6838.9 | -3404.5 | 6808.9 |
|  | **1+(1+Condition+Move\|ID)+Move*Condition*SequenceLenght** | **Yes** | **15** | **6775.5** | **-3372.8** | **6745.5** |
|  | 1+(1+Move+SequenceLenght\|ID)+Move*Condition*SequenceLenght | Yes | 15 | 6934.4 | -3452.2 | 6904.4 |

fMRI Results

Main effect of Self-generated > Instructed actions

The comparison between the self-generated and instructed actions showed activation of the inferior frontal gyrus in its opercularis portion, middle frontal gyrus, middle cingulum, pre-supplementary motor area, precentral gyrus, insula, superior and inferior parietal lobules, precuneus, lingual gyrus, calcarine scissure and cerebellum. See **Table S2**.

**Table S2. Results of the contrast Self-generated > Instructed actions. All results are p < 0.05, family-wise error (FWE) corrected at cluster-level; uncorrected p < 0.001 at voxel-level.**

| **Brain region (BA)** | **Peak MNI coordinates** | | | | | | | | | | | | **Cluster** | |
| --- | --- | --- | --- | --- | --- | --- | --- | --- | --- | --- | --- | --- | --- | --- |
|  | **Left hemisphere** | | | | | | **Right hemisphere** | | | | | |  |  |
|  | **x** | **y** | **z** | **T** | **Z** | **p(FWE-corr)** | **x** | **y** | **z** | **T** | **Z** | **p(FWE-corr)** | **p(FWE-corr)** | **Cluster size** |
| Insula (47) |  |  |  |  |  |  | 32 | 22 | 0 | 8.47 | 7.33 | p<0.001 | 0.001 | 638 |
| Superior Parietal Lobule (7) | -22 | -62 | 46 | 7.29 | 6.51 | p<0.001 | 20 | -62 | 60 | 7.12 | 6.39 | p<0.001 | 0.001 | 638 |
| Superior Parietal Lobule (5) |  |  |  |  |  |  | 14 | -58 | 64 | 7.2 | 6.45 | p<0.001 | 0.001 | 638 |
| Superior Parietal Lobule (5) |  |  |  |  |  |  | 20 | -56 | 62 | 7.08 | 6.36 | p<0.001 | 0.001 | 638 |
| Inferior Parietal Lobule (40) | -34 | -44 | 38 | 6.32 | 5.79 | p<0.001 | 38 | -40 | 44 | 7.76 | 6.85 | p<0.001 | 0.001 | 638 |
| Inferior Parietal Lobule (40) | -38 | -46 | 46 | 6.1 | 5.61 | p<0.001 |  |  |  |  |  |  | 0.001 | 638 |
| Inferior Parietal Lobule (7) | -26 | -52 | 42 | 6.22 | 5.71 | p<0.001 | 32 | -54 | 66 | 6.08 | 5.6 | 0.001 | 0.001 | 638 |
| Precuneus (5) | -12 | -60 | 54 | 7.69 | 6.8 | p<0.001 |  |  |  |  |  |  | 0.001 | 638 |
| Precuneus (5) | -10 | -56 | 66 | 5.38 | 5.03 | 0.008 |  |  |  |  |  |  | 0.001 | 638 |
| Precuneus |  |  |  |  |  |  | 10 | -60 | 50 | 7.05 | 6.34 | p<0.001 | 0.001 | 638 |
| Lingual gyrus (18) | -14 | -74 | -2 | 5.28 | 4.95 | 0.012 |  |  |  |  |  |  | 0.001 | 638 |
| Lingual gyrus (17) | -12 | -80 | 2 | 5.03 | 4.74 | 0.029 |  |  |  |  |  |  | 0.001 | 638 |
| Calcarine (17) | -10 | -78 | 10 | 4.86 | 4.6 | 0.052 |  |  |  |  |  |  | 0.001 | 638 |
| Middle Frontal gyrus (8) | -24 | 8 | 58 | 5.47 | 5.1 | 0.006 | 26 | 10 | 52 | 6.93 | 6.25 | p<0.001 | p<0.0001 | 2604 |
| Middle Frontal gyrus (8) | -26 | -4 | 50 | 4.99 | 4.7 | 0.033 |  |  |  |  |  |  | p<0.0001 | 2604 |
| Middle Cingulum (32) |  |  |  |  |  |  | 8 | 22 | 38 | 6.11 | 5.62 | p<0.001 | p<0.0001 | 2604 |
| Pre-supplementary motor area (6) | -8 | 14 | 46 | 5.02 | 4.73 | 0.03 |  |  |  |  |  |  | p<0.0001 | 2604 |
| Precentral gyrus (6) |  |  |  |  |  |  | 26 | -6 | 50 | 5.29 | 4.96 | 0.011 | p<0.0001 | 2604 |
| Insula (47) | -30 | 24 | 0 | 6.69 | 6.06 | p<0.001 |  |  |  |  |  |  | 0.006 | 408 |
| Middle Frontal gyrus (46) |  |  |  |  |  |  | 28 | 44 | 12 | 6.12 | 5.63 | p<0.001 | p<0.0001 | 1354 |
| Middle Frontal gyrus (46) |  |  |  |  |  |  | 34 | 34 | 26 | 6.08 | 5.59 | 0.001 | p<0.0001 | 1354 |
| Cerebellum_6 | -28 | -60 | -32 | 5.74 | 5.33 | 0.002 |  |  |  |  |  |  | 0.037 | 262 |
| Cerebellum_8 | -32 | -46 | -40 | 4.73 | 4.48 | 0.08 |  |  |  |  |  |  | 0.037 | 262 |
| Cerebellum_Vermis_8 | -4 | -74 | -28 | 5.61 | 5.22 | 0.003 |  |  |  |  |  |  | 0.002 | 515 |
| Cerebellum_Vermis_8 | 0 | -60 | -30 | 4.76 | 4.51 | 0.074 |  |  |  |  |  |  | 0.002 | 515 |
| Cerebellum_Vermis_8 | 0 | -60 | -36 | 4.71 | 4.47 | 0.085 |  |  |  |  |  |  | 0.002 | 515 |
| Cerebellum_Vermis_4_5 | 0 | -54 | -20 | 4.3 | 4.11 | 0.297 |  |  |  |  |  |  | 0.002 | 515 |
| Cerebellum_Vermis_4_5 | -2 | -52 | -16 | 4.09 | 3.92 | 0.485 |  |  |  |  |  |  | 0.002 | 515 |
| Inferior Frontal gyrus Opercularis pars (44) |  |  |  |  |  |  | 48 | 8 | 24 | 5.15 | 4.83 | 0.019 | 0.014 | 338 |
| Inferior Frontal gyrus Opercularis pars (44) | -46 | 6 | 26 | 4.35 | 4.15 | 0.259 |  |  |  |  |  |  | 0.031 | 277 |
| Precentral gyrus (6) | -42 | 0 | 32 | 4.3 | 4.11 | 0.297 |  |  |  |  |  |  | 0.031 | 277 |

*First move > other moves (Self-generated > Instructed actions)*

The comparison between first and other moves showed activation of the left inferior frontal gyrus partes opercularis and triangularis, left precental gyrus, left inferior temporal lobe, lingual and fusiform gyri, left middle and inferior occipital gyri, cerebellum, thalamus and caudate. See **Table S3**.

**Table S3. Results of the contrast first move > other moves. All results are p < 0.05, family-wise error (FWE) corrected at cluster-level; uncorrected p < 0.001 at voxel-level.**

| **Brain region (BA)** | **Peak MNI coordinates** | | | | | | | | | | | | **Cluster** | |
| --- | --- | --- | --- | --- | --- | --- | --- | --- | --- | --- | --- | --- | --- | --- |
|  | **Left hemisphere** | | | | | | **Right hemisphere** | | | | | |  |  |
|  | **x** | **y** | **z** | **T** | **Z** | **p(FWE-corr)** | **x** | **y** | **z** | **T** | **Z** | **p(FWE-corr)** | **p(FWE-corr)** | **Cluster size** |
| Inferior Temporal gyrus (20) | -52 | -52 | -16 | 3.28 | 3.19 | 0.997 |  |  |  |  |  |  | p<0.001 | 3589 |
| Lingual gyrus (18) | -16 | -88 | -8 | 7.45 | 6.63 | p<0.001 |  |  |  |  |  |  | p<0.001 | 3589 |
| Fusiform gyrus (37) | -32 | -42 | -18 | 3.76 | 3.63 | 0.818 |  |  |  |  |  |  | p<0.001 | 3589 |
|  | -38 | -48 | -18 | 3.58 | 3.46 | 0.939 |  |  |  |  |  |  | p<0.001 | 3589 |
| Fusiform gyrus (18) | -24 | -82 | -8 | 7.23 | 6.47 | p<0.001 |  |  |  |  |  |  | p<0.001 | 3589 |
| Middle Occipital gyrus (18) | -28 | -90 | 10 | 6.17 | 5.67 | p<0.001 |  |  |  |  |  |  | p<0.001 | 3589 |
| Middle Occipital gyrus (19) | -26 | -66 | 32 | 5.58 | 5.19 | 0.004 |  |  |  |  |  |  | p<0.001 | 3589 |
|  | -28 | -78 | 18 | 5.06 | 4.77 | 0.026 |  |  |  |  |  |  | p<0.001 | 3589 |
|  | -28 | -78 | 38 | 4.76 | 4.5 | 0.075 |  |  |  |  |  |  | p<0.001 | 3589 |
| Inferior Occipital gyrus (19) | -30 | -84 | -8 | 7.41 | 6.6 | p<0.001 |  |  |  |  |  |  | p<0.001 | 3589 |
| Inferior Occipital gyrus (37) |  |  |  |  |  |  | 40 | -64 | -12 | 4.62 | 4.39 | 0.114 | p<0.001 | 3589 |
| Cerebelum_6 | -38 | -40 | -26 | 4.31 | 4.11 | 0.29 |  |  |  |  |  |  | p<0.001 | 3589 |
| Fusiform gyrus (19) |  |  |  |  |  |  | 30 | -82 | -4 | 6.4 | 5.85 | p<0.001 | p<0.001 | 1711 |
| Lingual gyrus (18) |  |  |  |  |  |  | 14 | -78 | -2 | 4.34 | 4.15 | 0.263 | p<0.001 | 1711 |
| Lingual gyrus (19) |  |  |  |  |  |  | 18 | -68 | 2 | 3.28 | 3.19 | 0.997 | p<0.001 | 1711 |
| Middle Occipital gyrus (19) |  |  |  |  |  |  | 32 | -74 | 16 | 5.02 | 4.73 | 0.03 | p<0.001 | 1711 |
|  |  |  |  |  |  |  | 32 | -70 | 22 | 4.96 | 4.67 | 0.038 | p<0.001 | 1711 |
| Inferior Occipital gyrus (19) |  |  |  |  |  |  | 34 | -78 | -8 | 6.77 | 6.13 | p<0.001 | p<0.001 | 1711 |
| Thalamus |  |  |  |  |  |  | 10 | -6 | 14 | 3.31 | 3.22 | 0.996 | 0.001 | 636 |
| Caudate | -4 | 16 | 6 | 4.62 | 4.39 | 0.115 | 10 | 10 | 2 | 5.39 | 5.04 | 0.008 | 0.001 | 636 |
|  | -8 | 6 | 10 | 4.1 | 3.93 | 0.479 | 4 | 12 | -2 | 3.73 | 3.6 | 0.843 | 0.001 | 636 |
|  |  |  |  |  |  |  | 10 | -2 | 16 | 3.32 | 3.22 | 0.996 | 0.001 | 636 |
| Inferior Frontal gyrus triangularis pars | -36 | 22 | 20 | 5.12 | 4.81 | 0.021 |  |  |  |  |  |  | p<0.001 | 806 |
| Inferior Frontal gyrus triangularis pars (45) | -50 | 32 | 20 | 4 | 3.85 | 0.578 |  |  |  |  |  |  | p<0.001 | 806 |
| Inferior Frontal gyrus opercularis pars (44) | -52 | 20 | 34 | 3.89 | 3.74 | 0.7 |  |  |  |  |  |  | p<0.001 | 806 |
| Precentral gyrus | -40 | 2 | 28 | 5.28 | 4.95 | 0.012 |  |  |  |  |  |  | p<0.001 | 806 |
| Cerebelum_Crus2 |  |  |  |  |  |  | 32 | -72 | -36 | 3.93 | 3.78 | 0.657 | 0.013 | 347 |
|  |  |  |  |  |  |  | 34 | -70 | -44 | 3.88 | 3.73 | 0.711 | 0.013 | 347 |
|  |  |  |  |  |  |  | 34 | -76 | -38 | 3.78 | 3.65 | 0.805 | 0.013 | 347 |
| Cerebelum_Crus1 |  |  |  |  |  |  | 30 | -64 | -34 | 3.7 | 3.58 | 0.868 | 0.013 | 347 |
|  |  |  |  |  |  |  | 26 | -66 | -32 | 3.67 | 3.54 | 0.892 |  |  |

*Other moves > first move (Self-generated > Instructed actions)*

The comparison between other and first moves showed activation of the left Rolandic operculum, middle cingulum, precentral and postcentral gyri, superior parietal lobule and supramarginal gyrus, right superior and middle temporal gyrus. See **Table S4**.

**Table S4. Results of the contrast Other moves > first move. All results are p < 0.05, family-wise error (FWE) corrected at cluster-level; uncorrected p < 0.001 at voxel-level.**

| Brain region (BA) | Peak MNI coordinates | | | | | | | | | | | | Cluster | |
| --- | --- | --- | --- | --- | --- | --- | --- | --- | --- | --- | --- | --- | --- | --- |
|  | Left hemisphere | | | | | | Right hemisphere | | | | | |  |  |
|  | x | y | z | T | Z | p(FWE-corr) | x | y | z | T | Z | p(FWE-corr) | p(FWE-corr) | Cluster size |
| Rolandic Operculum | -46 | -28 | 18 | 5.48 | 5.11 | 0.005 |  |  |  |  |  |  | p<0.001 | 4690 |
|  | -48 | 0 | 6 | 5.36 | 5.02 | 0.009 |  |  |  |  |  |  | p<0.001 | 4690 |
| Middle Cingulum | -14 | -24 | 42 | 3.68 | 3.55 | 0.887 |  |  |  |  |  |  | p<0.001 | 4690 |
|  | -10 | -24 | 44 | 3.63 | 3.51 | 0.914 |  |  |  |  |  |  | p<0.001 | 4690 |
| Precentral gyrus (6) | -32 | -16 | 66 | 6.66 | 6.05 | p<0.001 |  |  |  |  |  |  | p<0.001 | 4690 |
|  | -20 | -16 | 76 | 3.83 | 3.69 | 0.756 |  |  |  |  |  |  | p<0.001 | 4690 |
| Precentral gyrus (4) | -40 | -24 | 62 | 8.08 | 7.07 | p<0.001 |  |  |  |  |  |  | p<0.001 | 4690 |
| Postcentral gyrus (3) | -52 | -22 | 48 | 6.48 | 5.91 | p<0.001 |  |  |  |  |  |  | p<0.001 | 4690 |
|  | -24 | -34 | 56 | 5.06 | 4.76 | 0.026 |  |  |  |  |  |  | p<0.001 | 4690 |
| Superior Parietal lobule (2) | -36 | -46 | 62 | 4.88 | 4.61 | 0.049 |  |  |  |  |  |  | p<0.001 | 4690 |
| Superior Parietal lobule (5) | -22 | -50 | 68 | 3.76 | 3.63 | 0.819 |  |  |  |  |  |  | p<0.001 | 4690 |
| Supramarginal gyrus | -56 | -24 | 20 | 6.75 | 6.11 | p<0.001 |  |  |  |  |  |  | p<0.001 | 4690 |
|  | -60 | -44 | 30 | 4.65 | 4.41 | 0.105 |  |  |  |  |  |  | p<0.001 | 4690 |
|  |  |  |  |  |  |  |  |  |  |  |  |  | p<0.001 |  |
| Supramarginal gyrus (40) |  |  |  |  |  |  | 52 | -34 | 28 | 5.58 | 5.19 | 0.004 | p<0.001 | 1840 |
|  |  |  |  |  |  |  | 62 | -36 | 30 | 5.57 | 5.19 | 0.004 | p<0.001 | 1840 |
| Superior Temporal gyrus (22) |  |  |  |  |  |  | 64 | -40 | 20 | 5.44 | 5.08 | 0.006 | p<0.001 | 1840 |
| Middle Temporal gyrus (37) |  |  |  |  |  |  | 52 | -54 | 4 | 5.37 | 5.02 | 0.008 | p<0.001 | 1840 |
|  |  |  |  |  |  |  | 40 | -48 | 2 | 4.34 | 4.14 | 0.267 | p<0.001 | 1840 |
|  |  |  |  |  |  |  | 58 | -66 | 10 | 4.32 | 4.13 | 0.28 | p<0.001 | 1840 |
|  |  |  |  |  |  |  | 60 | -50 | 10 | 4.12 | 3.95 | 0.454 | p<0.001 | 1840 |
|  |  |  |  |  |  |  | 54 | -68 | -4 | 3.63 | 3.51 | 0.917 | p<0.001 | 1840 |
|  |  |  |  |  |  |  |  |  |  |  |  |  |  |  |
| Parietal Operculum |  |  |  |  |  |  | 32 | -34 | 24 | 4.47 | 4.26 | 0.181 | 0.009 | 374 |
|  |  |  |  |  |  |  | 30 | -30 | 24 | 3.81 | 3.68 | 0.775 | 0.009 | 374 |
| Corpus Callosum |  |  |  |  |  |  | 8 | -24 | 26 | 3.58 | 3.47 | 0.938 | 0.009 | 374 |
|  |  |  |  |  |  |  | 14 | -22 | 30 | 3.47 | 3.36 | 0.976 | 0.009 | 374 |
|  |  |  |  |  |  |  | 12 | -26 | 26 | 3.34 | 3.25 | 0.994 | 0.009 | 374 |

*4 moves > 2 moves problems (Self-generated > Instructed actions)*

The comparison between problems with 4 moves and problems of 2 moves showed activation of the left superior frontal gyrus, right middle frontal gyrus, middle cingulum, left pre-supplementary motor area, left superior parietal lobule and precuneus. See **Table S5**.

**Table S5. Results of the contrast 4 moves > 2 moves problems. All results are p < 0.05, family-wise error (FWE) corrected at cluster-level; uncorrected p < 0.001 at voxel-level.**

| **Brain region (BA)** | **Peak MNI coordinates** | | | | | | | | | | | | **Cluster** | |
| --- | --- | --- | --- | --- | --- | --- | --- | --- | --- | --- | --- | --- | --- | --- |
|  | **Left hemisphere** | | | | | | **Right hemisphere** | | | | | |  |  |
|  | **x** | **y** | **z** | **T** | **Z** | **p(FWE-corr)** | **x** | **y** | **z** | **T** | **Z** | **p(FWE-corr)** | **p(FWE-corr)** | **Cluster size** |
| Superior Frontal gyrus (6) | -20 | 6 | 58 | 6.71 | 6.08 | p<0.001 |  |  |  |  |  |  | p<0.001 | 1314 |
|  | -20 | 4 | 66 | 6.25 | 5.73 | p<0.001 |  |  |  |  |  |  | p<0.001 | 1314 |
|  | -22 | -2 | 52 | 6.03 | 5.55 | 0.001 |  |  |  |  |  |  | p<0.001 | 1314 |
| Middle Cingulum (32) | -18 | 10 | 42 | 3.87 | 3.72 | 0.722 |  |  |  |  |  |  | p<0.001 | 1314 |
|  | -14 | 18 | 38 | 3.7 | 3.57 | 0.868 |  |  |  |  |  |  | p<0.001 | 1314 |
|  |  |  |  |  |  |  |  |  |  |  |  |  | p<0.001 | 1314 |
| Supplementary Motor Area (6) | -16 | -4 | 58 | 5.93 | 5.48 | 0.001 |  |  |  |  |  |  | p<0.001 | 1314 |
|  | -8 | 6 | 52 | 4.94 | 4.66 | 0.04 |  |  |  |  |  |  | p<0.001 | 1314 |
|  | -10 | 12 | 48 | 4.77 | 4.52 | 0.071 |  |  |  |  |  |  | p<0.001 | 1314 |
| Superior Parietal lobule (7) | -26 | -58 | 46 | 3.61 | 3.49 | 0.927 |  |  |  |  |  |  | p<0.001 | 1667 |
|  |  |  |  |  |  |  |  |  |  |  |  |  | p<0.001 | 1667 |
| Precuneus (5) | -12 | -58 | 54 | 6.62 | 6.02 | p<0.001 | 8 | -54 | 52 | 5.22 | 4.9 | 0.015 | p<0.001 | 1667 |
|  | -4 | -56 | 50 | 5.64 | 5.24 | 0.003 |  |  |  |  |  |  | p<0.001 | 1667 |
| Middle Frontal gyrus (8) |  |  |  |  |  |  | 26 | 8 | 50 | 5.28 | 4.95 | 0.012 | p<0.001 | 974 |
| Middle Frontal gyrus (6) |  |  |  |  |  |  | 28 | 4 | 50 | 5.13 | 4.82 | 0.02 | p<0.001 | 974 |
|  |  |  |  |  |  |  | 18 | 2 | 54 | 4.9 | 4.63 | 0.046 | p<0.001 | 974 |
|  |  |  |  |  |  |  | 20 | 4 | 68 | 4.59 | 4.36 | 0.129 | p<0.001 | 974 |
| Middle Cingulum (32) |  |  |  |  |  |  | 14 | 16 | 44 | 3.41 | 3.3 | 0.987 | p<0.001 | 974 |

*Effect of “sequence length”: 2 moves > 4 moves problems (Self-generated > Instructed actions)*

The comparison between problems with 2 moves and problems of 4 moves showed activation of the right cuneus, Rolandic operculum, insula, right supramarginal gyrus, right superior temporal gyrus, left calcarine scissure, left corpus callosum and right corona radiata. See **Table S6**.

**Table S6. Results of the contrast 2 moves > 4 moves problems. All results are p < 0.05, family-wise error (FWE) corrected at cluster-level; uncorrected p < 0.001 at voxel-level.**

| Brain region (BA) | Peak MNI coordinates | | | | | | | | | | | | Cluster | |
| --- | --- | --- | --- | --- | --- | --- | --- | --- | --- | --- | --- | --- | --- | --- |
|  | Left hemisphere | | | | | | Right hemisphere | | | | | |  |  |
|  | x | y | z | T | Z | p(FWE-corr) | x | y | z | T | Z | p(FWE-corr) | p(FWE-corr) | Cluster size |
| Cuneus |  |  |  |  |  |  | 4 | -84 | 34 | 6.21 | 5.7 | p<0.0001 | 0.003 | 465 |
| Rolandic Operculum | -40 | -26 | 18 | 5.57 | 5.18 | 0.004 |  |  |  |  |  |  | p<0.001 | 2067 |
|  | -50 | -20 | 16 | 5.53 | 5.15 | 0.005 |  |  |  |  |  |  | p<0.001 | 2067 |
|  | -40 | -20 | 16 | 5.33 | 4.99 | 0.01 |  |  |  |  |  |  | p<0.001 | 2067 |
|  | -50 | -14 | 14 | 4.73 | 4.49 | 0.08 |  |  |  |  |  |  | p<0.001 | 2067 |
|  | -60 | 2 | 8 | 4.11 | 3.94 | 0.467 |  |  |  |  |  |  | p<0.001 | 2067 |
| Corpus Callosum | -18 | -46 | 20 | 4.94 | 4.66 | 0.04 |  |  |  |  |  |  | p<0.001 | 2067 |
|  | -18 | -30 | 22 | 4.86 | 4.59 | 0.053 |  |  |  |  |  |  | p<0.001 | 2067 |
| Insula | -38 | -12 | 14 | 4.54 | 4.31 | 0.15 |  |  |  |  |  |  | p<0.001 | 2067 |
| Calcarine scissure (17) | -24 | -56 | 10 | 3.96 | 3.81 | 0.627 |  |  |  |  |  |  | p<0.001 | 2067 |
| Corona Radiata |  |  |  |  |  |  | 20 | -36 | 30 | 5.26 | 4.93 | 0.013 | 0.004 | 436 |
| Rolandic Operculum |  |  |  |  |  |  | 44 | -2 | 18 | 4.39 | 4.19 | 0.232 | p<0.001 | 675 |
|  |  |  |  |  |  |  | 46 | -6 | 18 | 4.28 | 4.09 | 0.31 | p<0.001 | 675 |
|  |  |  |  |  |  |  | 50 | -14 | 20 | 4.27 | 4.08 | 0.318 | p<0.001 | 675 |
|  |  |  |  |  |  |  | 56 | -12 | 14 | 4.24 | 4.05 | 0.345 | p<0.001 | 675 |
|  |  |  |  |  |  |  | 52 | -14 | 16 | 4.21 | 4.03 | 0.371 | p<0.001 | 675 |
|  |  |  |  |  |  |  | 52 | 0 | 10 | 3.54 | 3.42 | 0.957 | p<0.001 | 675 |
|  |  |  |  |  |  |  | 38 | -4 | 20 | 3.5 | 3.39 | 0.969 | p<0.001 | 675 |
|  |  |  |  |  |  |  | 44 | -26 | 20 | 3.5 | 3.39 | 0.969 | p<0.001 | 675 |
| Insula |  |  |  |  |  |  | 42 | -12 | 12 | 4.41 | 4.2 | 0.219 | p<0.001 | 675 |
| Supramarginal gyrus |  |  |  |  |  |  | 56 | -24 | 20 | 4.37 | 4.17 | 0.246 | p<0.001 | 675 |
| Superior Temporal gyrus |  |  |  |  |  |  | 46 | -26 | 16 | 3.45 | 3.35 | 0.98 | p<0.001 | 675 |

*Interaction between “move” and “sequence length” (Self-generated > Instructed actions)*

The interaction between the factors “move” and “sequence length” showed activation of the bilateral superior and middle frontal gyrus, left middle cingulum, right supplementary motor area, bilateral precentral gyrus, left postcentral gyrus, bilateral superior and inferior parietal lobule, and precuneus. See **Table S7**.

**Table S7. Results of the Interaction between “move” and “sequence length”. All results are p < 0.05, family-wise error (FWE) corrected at cluster-level; uncorrected p < 0.001 at voxel-level.**

| Brain region (BA) | Peak MNI coordinates | | | | | | | | | | | | Cluster | |
| --- | --- | --- | --- | --- | --- | --- | --- | --- | --- | --- | --- | --- | --- | --- |
|  | Left hemisphere | | | | | | Right hemisphere | | | | | |  |  |
|  | x | y | z | T | Z | p(FWE-corr) | x | y | z | T | Z | p(FWE-corr) | p(FWE-corr) | Cluster size |
| Postcentral gyrus (40) | -40 | -34 | 48 | 4.47 | 4.25 | 0.185 |  |  |  |  |  |  | p<0.001 | 4780 |
| Postcentral gyrus (2) | -40 | -32 | 44 | 4.37 | 4.17 | 0.241 |  |  |  |  |  |  | p<0.001 | 4780 |
| Superior parietal lobule (5) | -18 | -58 | 66 | 4.72 | 4.48 | 0.084 | 22 | -50 | 64 | 4.49 | 4.28 | 0.171 | p<0.001 | 4780 |
| Superior parietal lobule (7) | -24 | -48 | 50 | 4.67 | 4.43 | 0.097 | 16 | -72 | 54 | 4.67 | 4.43 | 0.097 | p<0.001 | 4780 |
|  |  |  |  |  |  |  | 16 | -66 | 58 | 4.46 | 4.25 | 0.189 | p<0.001 | 4780 |
| Inferior parietal lobule (40) | -32 | -46 | 44 | 5.32 | 4.98 | 0.01 |  |  |  |  |  |  | p<0.001 | 4780 |
|  | -32 | -52 | 54 | 4.99 | 4.7 | 0.034 |  |  |  |  |  |  | p<0.001 | 4780 |
| Inferior parietal lobule (2) | -40 | -32 | 34 | 4.88 | 4.61 | 0.05 |  |  |  |  |  |  | p<0.001 | 4780 |
|  | -44 | -32 | 36 | 4.66 | 4.42 | 0.102 |  |  |  |  |  |  | p<0.001 | 4780 |
| Precuneus (5) | -12 | -62 | 56 | 6.57 | 5.98 | p<0.001 | 8 | -54 | 54 | 4.6 | 4.37 | 0.125 | p<0.001 | 4780 |
| Superior occipital gyrus (7) |  |  |  |  |  |  | 22 | -62 | 46 | 4.37 | 4.17 | 0.242 | p<0.001 | 4780 |
| Superior frontal gyrus (6) | -20 | 2 | 68 | 5.42 | 5.06 | 0.007 |  |  |  |  |  |  | p<0.001 | 1624 |
| Middle frontal gyrus (6) | -26 | -6 | 52 | 6.28 | 5.75 | p<0.001 |  |  |  |  |  |  | p<0.001 | 1624 |
| Middle cingulum | -16 | 4 | 44 | 4.46 | 4.25 | 0.186 |  |  |  |  |  |  | p<0.001 | 1624 |
| Supplemetary motor area (6) | -8 | 6 | 50 | 5.12 | 4.81 | 0.021 |  |  |  |  |  |  | p<0.001 | 1624 |
| Precentral gyrus (6) | -40 | -6 | 44 | 3.3 | 3.21 | 0.997 |  |  |  |  |  |  | p<0.001 | 1624 |
| Superior frontal gyrus (6) |  |  |  |  |  |  | 24 | -10 | 54 | 4.94 | 4.66 | 0.04 | p<0.001 | 985 |
| Superior frontal gyrus |  |  |  |  |  |  | 20 | 4 | 52 | 3.83 | 3.69 | 0.763 | p<0.001 | 985 |
| Middle frontal gyrus (8) |  |  |  |  |  |  | 24 | 8 | 48 | 4.16 | 3.99 | 0.412 | p<0.001 | 985 |
| Supplementary motor area (6) |  |  |  |  |  |  | 14 | 8 | 54 | 3.53 | 3.42 | 0.959 | p<0.001 | 985 |
| Precentral gyrus (6) |  |  |  |  |  |  | 28 | -4 | 50 | 5.02 | 4.73 | 0.03 | p<0.001 | 985 |
|  |  |  |  |  |  |  | 22 | -14 | 54 | 4.93 | 4.65 | 0.041 | p<0.001 | 985 |
|  |  |  |  |  |  |  | 26 | -6 | 54 | 4.86 | 4.59 | 0.053 | p<0.001 | 985 |
|  |  |  |  |  |  |  | 26 | -8 | 50 | 4.73 | 4.48 | 0.08 | p<0.001 | 985 |
| Inferior parietal lobule |  |  |  |  |  |  | 46 | -36 | 54 | 3.55 | 3.44 | 0.95 | 0.001 | 562 |
| Supramarginal gyrus (40) |  |  |  |  |  |  | 34 | -40 | 46 | 4.75 | 4.5 | 0.075 | 0.001 | 562 |

Generalised Psychophysiological interaction analysis.

*First move > other moves*

The comparison between first and other moves showed stronger pre-SMA connectivity with the right superior frontal gyrus, left inferior frontal gyrus triangularis and opercularis parts, right anterior cingulum, bilateral middle cingulum, bilateral superior temporal gyrus, left temporal gyrus, bilateral superior and middle temporal gyri, bilateral insula, left hippocampus. See **Table S8**.

**Table S8. Pre-SMA connectivity patterns: results of the contrast first move > other moves. All results are p < 0.05, family-wise error (FWE) corrected at cluster-level; uncorrected p < 0.001 at voxel-level.**

| Brain region (BA) | Peak MNI coordinates | | | | | | | | | | | | Cluster | |
| --- | --- | --- | --- | --- | --- | --- | --- | --- | --- | --- | --- | --- | --- | --- |
|  | Left hemisphere | | | | | | Right hemisphere | | | | | |  |  |
|  | x | y | z | T | Z | p(FWE-corr) | x | y | z | T | Z | p(FWE-corr) | p(FWE-corr) | Cluster size |
| Superior Frontal Gyrus (32) |  |  |  |  |  |  | 4 | 28 | 50 | 4.84 | 4.57 | 0.175 | p<0.001 | 1663 |
|  |  |  |  |  |  |  | 4 | 42 | 36 | 4.25 | 4.07 | 0.728 | p<0.001 | 1663 |
| Superior Frontal Gyrus (9) |  |  |  |  |  |  | 10 | 50 | 38 | 4.19 | 4.01 | 0.798 | p<0.001 | 1663 |
|  |  |  |  |  |  |  | 8 | 46 | 36 | 4.09 | 3.93 | 0.881 | p<0.001 | 1663 |
| Anterior Cingulum (32) | -2 | 18 | 20 | 4.63 | 4.4 | 0.319 | 4 | 38 | 30 | 4.5 | 4.28 | 0.451 | p<0.001 | 1663 |
|  |  |  |  |  |  |  | 0 | 16 | 24 | 4.41 | 4.21 | 0.549 | p<0.001 | 1663 |
|  |  |  |  |  |  |  | 0 | 40 | 14 | 4.31 | 4.11 | 0.669 | p<0.001 | 1663 |
|  |  |  |  |  |  |  | 0 | 42 | 10 | 4.24 | 4.05 | 0.745 | p<0.001 | 1663 |
|  |  |  |  |  |  |  | 4 | 42 | 12 | 3.99 | 3.84 | 0.943 | p<0.001 | 1663 |
| Anterior Cingulum (11) |  |  |  |  |  |  | 2 | 40 | -2 | 4.09 | 3.92 | 0.885 | p<0.001 | 1663 |
| Middle Cingulum (24) |  |  |  |  |  |  | 4 | 8 | 34 | 4.57 | 4.35 | 0.372 | p<0.001 | 1663 |
|  |  |  |  |  |  |  | 0 | 24 | 32 | 4.5 | 4.29 | 0.444 | p<0.001 | 1663 |
|  |  |  |  |  |  |  | 4 | 6 | 28 | 4.59 | 4.36 | 0.355 | p<0.001 | 1663 |
| Corpus Callosum |  |  |  |  |  |  | 4 | 20 | 14 | 4.48 | 4.26 | 0.475 | p<0.001 | 1663 |
|  |  |  |  |  |  |  | 4 | 24 | 12 | 4.32 | 4.13 | 0.649 | p<0.001 | 1663 |
| Superior Temporal Gyrus (42) | -58 | -44 | 24 | 3.23 | 3.14 | 1.00 |  |  |  |  |  |  | 0.012 | 178 |
| Middle Temporal Gyrus (22) | -52 | -50 | 20 | 4.52 | 4.3 | 0.432 |  |  |  |  |  |  | 0.012 | 178 |
|  |  |  |  |  |  |  |  |  |  |  |  |  | 0.012 | 178 |
|  |  |  |  |  |  |  |  |  |  |  |  |  | 0.012 | 178 |
| Rolandic Operculum | 42 | -4 | -12 | 4.01 | 3.85 | 0.935 |  |  |  |  |  |  | p<0.001 | 697 |
| Superior Temporal Gyrus |  |  |  |  |  |  | 56 | -8 | -4 | 4.5 | 4.29 | 0.445 | p<0.001 | 697 |
|  |  |  |  |  |  |  | 52 | -10 | -4 | 4.42 | 4.21 | 0.541 | p<0.001 | 697 |
|  |  |  |  |  |  |  | 48 | -14 | -6 | 4.34 | 4.15 | 0.625 | p<0.001 | 697 |
|  |  |  |  |  |  |  | 42 | -20 | -4 | 4.01 | 3.85 | 0.936 | p<0.001 | 697 |
|  |  |  |  |  |  |  | 40 | -24 | 2 | 3.95 | 3.8 | 0.962 | p<0.001 | 697 |
|  |  |  |  |  |  |  | 44 | -16 | -6 | 4.17 | 3.99 | 0.816 | p<0.001 | 697 |
| Insula |  |  |  |  |  |  | 36 | 8 | 2 | 4.34 | 4.15 | 0.628 | p<0.001 | 697 |
|  |  |  |  |  |  |  | 48 | 8 | 4 | 4.32 | 4.13 | 0.65 | p<0.001 | 697 |
|  |  |  |  |  |  |  | 44 | 6 | 8 | 4.19 | 4.02 | 0.79 | p<0.001 | 697 |
|  |  |  |  |  |  |  | 42 | 6 | 2 | 4.04 | 3.88 | 0.917 | p<0.001 | 697 |
|  |  |  |  |  |  |  | 50 | 6 | -2 | 3.6 | 3.48 | 1 | p<0.001 | 697 |
|  |  |  |  |  |  |  | 36 | 16 | -4 | 3.47 | 3.36 | 1 | p<0.001 | 697 |
| Putamen | 34 | 10 | -6 | 3.8 | 3.67 | 0.992 |  |  |  |  |  |  | p<0.001 | 697 |
| Putamen | 34 | -22 | -2 | 3.67 | 3.55 | 0.999 |  |  |  |  |  |  | p<0.001 | 697 |
| Middle Cingulum (23) | -6 | -30 | 40 | 4.35 | 4.15 | 0.619 | 8 | -30 | 30 | 4.18 | 4 | 0.806 | p<0.001 | 554 |
|  | -2 | -32 | 40 | 4.24 | 4.05 | 0.748 | 6 | -26 | 30 | 4.18 | 4 | 0.81 | p<0.001 | 554 |
|  |  |  |  |  |  |  | 4 | -36 | 44 | 4.08 | 3.91 | 0.89 | p<0.001 | 554 |
|  |  |  |  |  |  |  | 2 | -36 | 38 | 4.03 | 3.87 | 0.921 | p<0.001 | 554 |
|  |  |  |  |  |  |  | 4 | -20 | 34 | 3.93 | 3.78 | 0.969 | p<0.001 | 554 |
|  |  |  |  |  |  |  | 2 | -28 | 44 | 3.89 | 3.75 | 0.978 | p<0.001 | 554 |
|  |  |  |  |  |  |  | 2 | -20 | 38 | 3.73 | 3.6 | 0.997 | p<0.001 | 554 |
|  |  |  |  |  |  |  | 6 | -34 | 32 | 3.72 | 3.59 | 0.998 | p<0.001 | 554 |
|  |  |  |  |  |  |  | 0 | -28 | 34 | 3.69 | 3.56 | 0.999 | p<0.001 | 554 |
|  |  |  |  |  |  |  | 2 | -14 | 38 | 3.55 | 3.44 | 1 | p<0.001 | 554 |
|  |  |  |  |  |  |  | 8 | -20 | 36 | 3.4 | 3.3 | 1 | p<0.001 | 554 |
| Superior Temporal Gyrus | -44 | -10 | -8 | 4.32 | 4.13 | 0.648 |  |  |  |  |  |  | 0.001 | 298 |
|  | -42 | -6 | -10 | 4.06 | 3.9 | 0.902 |  |  |  |  |  |  | 0.001 | 298 |
|  | -48 | -6 | 0 | 3.48 | 3.37 | 1.00 |  |  |  |  |  |  | 0.001 | 298 |
|  | -36 | -26 | 8 | 4.09 | 3.92 | 0.884 |  |  |  |  |  |  | 0.001 | 298 |
|  | -36 | -26 | 2 | 3.5 | 3.39 | 1.00 |  |  |  |  |  |  | 0.001 | 298 |
| Insula | -38 | -12 | -2 | 4.05 | 3.89 | 0.912 |  |  |  |  |  |  | 0.001 | 298 |
| Insula | -36 | -18 | 8 | 3.72 | 3.59 | 0.998 |  |  |  |  |  |  | 0.001 | 298 |
| Hippocampus | -36 | -16 | -10 | 3.67 | 3.55 | 0.999 |  |  |  |  |  |  | 0.001 | 298 |
| Inferior Frontal gyrus triangularis pars (45) | 54 | 32 | 16 | 4.05 | 3.89 | 0.91 |  |  |  |  |  |  | p<0.001 | 463 |
|  | 56 | 24 | 20 | 3.81 | 3.67 | 0.991 |  |  |  |  |  |  | p<0.001 | 463 |
|  | 48 | 26 | 0 | 3.76 | 3.62 | 0.996 |  |  |  |  |  |  | p<0.001 | 463 |
|  | 44 | 28 | 8 | 3.66 | 3.53 | 0.999 |  |  |  |  |  |  | p<0.001 | 463 |
|  | 42 | 22 | 6 | 3.65 | 3.53 | 0.999 |  |  |  |  |  |  | p<0.001 | 463 |
|  | 48 | 22 | 16 | 3.62 | 3.5 | 1.00 |  |  |  |  |  |  | p<0.001 | 463 |
|  | 56 | 24 | 6 | 3.58 | 3.46 | 1.00 |  |  |  |  |  |  | p<0.001 | 463 |
|  | 50 | 28 | 4 | 3.47 | 3.36 | 1.00 |  |  |  |  |  |  | p<0.001 | 463 |
|  | 42 | 22 | 16 | 3.41 | 3.31 | 1.00 |  |  |  |  |  |  | p<0.001 | 463 |
| Inferior Frontal opercularis pars | 58 | 18 | 8 | 3.43 | 3.33 | 1.00 |  |  |  |  |  |  | p<0.001 | 463 |
|  | 58 | 20 | 16 | 3.93 | 3.78 | 0.967 |  |  |  |  |  |  | p<0.001 | 463 |

*Other moves > first move*

The comparison between first and other moves showed stronger pre-SMA connectivity with the left superior parietal lobule and precuneus. See **Table S9.**

**Table S9. Pre-SMA connectivity patterns: results of the contrast other moves > first move. All results are p < 0.05, family-wise error (FWE) corrected at cluster-level; uncorrected p < 0.001 at voxel-level.**

| Brain region (BA) | Peak MNI coordinates | | | | | | | | | | | | Cluster | |
| --- | --- | --- | --- | --- | --- | --- | --- | --- | --- | --- | --- | --- | --- | --- |
|  | Left hemisphere | | | | | | Right hemisphere | | | | | |  |  |
|  | x | y | z | T | Z | p(FWE-corr) | x | y | z | T | Z | p(FWE-corr) | p(FWE-corr) | Cluster size |
| Superior Parietal Lobule (7) | -26 | -54 | 68 | 3.72 | 3.59 | p<0.001 |  |  |  |  |  |  | 0.998 | 437 |
|  | -20 | -66 | 56 | 3.66 | 3.53 | p<0.001 |  |  |  |  |  |  | 0.999 | 437 |
| Precuneus (7) | -12 | -60 | 64 | 4.4 | 4.2 | p<0.001 |  |  |  |  |  |  | 0.556 | 437 |
| Precuneus (5) | -8 | -58 | 66 | 4.35 | 4.16 | p<0.001 |  |  |  |  |  |  | 0.615 | 437 |

*4 moves > 2 moves problems*

The comparison between problems with 4 moves and problems of 2 moves showed no suprathreshold clusters significantly connected with the region of interest.

*2 moves > 4 moves problems*

The comparison between problems with 2 moves and problems of 4 moves showed a stronger connectivity between pre-SMA and the left inferior frontal gyrus opercularis pars, the right cerebellum also encompassing the right fusiform gyrus. See **Table S10**.

**Table S10. Pre-SMA connectivity patterns: results of the contrast 2 moves > 4 moves problems. Results are p < 0.05, family-wise error (FWE) corrected at cluster-level; uncorrected p < 0.001 at voxel-level.**

| Brain region (BA) | Peak MNI coordinates | | | | | | | | | | | | Cluster | |
| --- | --- | --- | --- | --- | --- | --- | --- | --- | --- | --- | --- | --- | --- | --- |
|  | Left hemisphere | | | | | | Right hemisphere | | | | | |  |  |
|  | x | y | z | T | Z | p(FWE-corr) | x | y | z | T | Z | p(FWE-corr) | p(FWE-corr) | Cluster size |
| Inferior frontal gyrus opercularis pars | -32 | 30 | -18 | 5.41 | 5.06 | 0.024 |  |  |  |  |  |  | 0.034 | 142 |
| Cerebellum |  |  |  |  |  |  | 30 | -44 | -20 | 4.37 | 4.17 | 0.598 | 0.027 | 150 |
|  |  |  |  |  |  |  | 30 | -54 | -20 | 3.79 | 3.66 | 0.994 | 0.027 | 150 |
|  |  |  |  |  |  |  | 34 | -50 | -26 | 3.39 | 3.29 | 1.00 | 0.027 | 150 |
| Fusiform gyrus (37) |  |  |  |  |  |  | 28 | -38 | -16 | 3.69 | 3.56 | 0.999 | 0.027 | 150 |
|  |  |  |  |  |  |  | 34 | -36 | -12 | 3.61 | 3.49 | 1.00 | 0.027 | 150 |
|  |  |  |  |  |  |  | 34 | -58 | -18 | 3.4 | 3.3 | 1.00 | 0.027 | 150 |
|  |  |  |  |  |  |  | 24 | -48 | -14 | 3.39 | 3.29 | 1.00 | 0.027 | 150 |
|  |  |  |  |  |  |  | 30 | -40 | -12 | 3.29 | 3.2 | 1.00 | 0.027 | 150 |

*Interaction between “move” and “sequence length”*

The interaction between the factors “move” and “sequence length” showed that pre-SMA was significantly stronger connected to the left superior frontal gyrus and right middle cingulum during the first move of a 4 moves problem. See Table S11 and Figure S2.

**Table S11. Pre-SMA connectivity patterns: results of the interaction between “move” and “sequence length”. Results are p < 0.05, family-wise error (FWE) corrected at cluster-level; uncorrected p < 0.001 at voxel-level.**

| Brain region (BA) | Peak MNI coordinates | | | | | | | | | | | | Cluster | |
| --- | --- | --- | --- | --- | --- | --- | --- | --- | --- | --- | --- | --- | --- | --- |
|  | Left hemisphere | | | | | | Right hemisphere | | | | | |  |  |
|  | x | y | z | T | Z | p(FWE-corr) | x | y | z | T | Z | p(FWE-corr) | p(FWE-corr) | Cluster size |
| Superior Frontal Gyrus (32) | -4 | 36 | 38 | 4.44 | 4.23 | 0.515 |  |  |  |  |  |  | p<0.0001 | 337 |
|  | -10 | 30 | 34 | 3.96 | 3.81 | 0.955 |  |  |  |  |  |  | p<0.0001 | 337 |
| Middle Cingulum (32) |  |  |  |  |  |  | 6 | 38 | 32 | 3.79 | 3.66 | 0.994 | p<0.0001 |  |

Meta-analysis of neuroimaging studies of volition

*Data collection and preparation*

Records were retrieved through the following queries in PubMed: [Voluntary action OR Intentional action OR willed action OR internally-generated action] AND [fMRI OR PET]. After the removal of duplicates, the initial set of studies included 350 papers, updated to August 2021. Papers were included when fulfilling the following inclusion criteria:

- Populations involved: healthy adult participants (no minimum sample size)
- Anatomical conventions: only data reported using MNI or Talairach coordinates, from both ROI (region of interest) and whole-brain analyses were included in the final dataset.
- Activation protocols: we considered only experimental paradigms in which actions were characterized by a clear volition component: namely, action selection between different action alternatives (equivalent to the so-called “what component” of voluntary action, Brass and Haggard, 2008), multiple execution timing ( “when component”), or between free choice of acting vs. inhibiting the action ( “whether component”) and instructed choice. Data resulting from the comparison between action execution and inhibition were not included.
- Statistical comparisons (linear contrasts) included: intentional action > stimulus-driven action. Data describing “deactivations” (intentional action < stimulus-driven action) were beyond the scope of the present review and were not included in the analysis. Data reporting null results (absence of a significant effect of intentional action > stimulus-driven action) were not considered; importantly, as for most meta-analyses, this choice may represent a source of bias as it may obscure the absence of an effect at all.
- Only data from univariate analyses with a minimum threshold of *p* < 0.05 uncorrected.

The final data set included 24 studies, comprising 34 statistical comparisons, and reporting 347 activation foci. See **Table S12**.

**Table S12. Neuroimaging studies on internally-generated actions.**

| **Authors and year** | **Technique** | **Contrast of interest** | **Main results** |
| --- | --- | --- | --- |
|  |  |  |  |
| **Deiber et al. (1991)** | PET | Freely determined movement vs. fixed movement | Greater activation in the supplementary motor cortex when subjects performed tasks based on internal rather than external cues. |
| **Frith et al. (1991)** | PET | Intention tasks (verbal & motor modality) vs. routine tasks. | Willed actions in the two modalities (speaking a word or lifting a finger) were associated with increased blood flow in the dorsolateral prefrontal cortex. |
| **Jahanshahi et al. (1995)** | PET | Self-initiated vs. externally triggered movements. | Left primary sensorimotor cortex, thalamus and putamen, bilateral SMA, anterior cingulate, lateral premotor cortex, insular cortex parietal area and right DLPFC were significantly activated during the self-initiated movements relative to rest. Greater activation of the right DLPFC during the self-initiated movements vs. externally triggered movements. |
| **Hyder et al. (1997)** | fMRI | Free-determined movement vs. fixed movement. | Greater activation in the middle frontal gyrus, superior frontal sulcus, right superior frontal gyrus and right anterior cingulate when subjects performed tasks based on internal than external cues. |
| **Jenkins et al. (2000)** | PET | Intentional finger movements vs. fixed. | Greater activation in the supplementary motor cortex when subjects performed tasks based on internal rather than external cues. |
| **Cunnington et al. (2002)** | fMRI | Self-initiated vs. externally triggered movements. | Self-initiated movement activated the medial motor areas, the superior parietal lobule, the insula cortex and the basal ganglia. The timing of the hemodynamic response within the pre-SMA was significantly earlier for self-initiated compared with externally triggered movements. |
| **Hunter et al. (2003)** | fMRI | Intentional bottom presses vs. implicit baseline. | Significant activation in the bilateral prefrontal cortex, left primary motor cortex, and supplementary motor cortex when subjects performed spontaneous action (implicit baseline). |
| **Blouin et al. (2004)** | PET | Self-initiated versus externally triggered condition. | Greater activation in the left premotor cortex, left dorsolateral prefrontal cortex, left cerebellum, right pre-supplementary motor area and the right parietal lobule in self-initiated synchronized movements compared to the externally triggered movements. |
| **Lau et al. (Lau et al., 2004)** | fMRI | Intentional target choice vs. specified target choice. | Greater activation in the pre-supplementary motor cortex when subjects performed tasks based on internal than external cues. |
| **Wiese et al. (2005)** | fMRI | Self-initiated in comparison to externally triggered movements. | Significantly enhanced activity in the left SMA, the left pre- and sensorimotor cortex, the right putamen, the left anterior cingulate gyrus, and the left inferior parietal lobe during self-initiated compared to externally triggered movements. |
|  |  |  |  |
| **Rowe et al. (2005)** | fMRI | Free selection of action vs. externally specified action. | Greater activation in the left middle frontal gyrus, inferior parietal cortex and right middle frontal gyrus when subjects performed tasks based on free selection of movements than externally specified actions. |
| **Forstmann et al. (2006)** | fMRI | Intentional choice condition vs. forced condition. | Greater activation in the rostral cingulate zone, the superior parietal lobule and the intraparietal sulcus when subjects performed tasks based on internal than external cues. |
|  |  |  |  |
| **van Eimeren et al. (2006)** | fMRI | Response selection vs. no response selection. | Greater activation in the anterior part of rostral dorsal premotor cortex, the rostral cingulate, supplementary motor area, and the right dorsolateral prefrontal cortex when subjects performed tasks based on internal rather than external cues. |
| **Thobois et al. (2007)** | PET | Self-initiated movements vs. externally cued. | Self-initiated versus externally cued activated the prefrontal cortical areas. |
| **Mueller et al. (2007)** | fMRI | Intentionally vs. externally selected actions. | Greater activation in the rostral cingulate zone when subjects performed tasks based on internal than external cues. |
|  |  |  |  |
| **Boecker et al. (2008)** | fMRI | Internally initiated condition vs. externally triggered condition. | Greater activity in frontal regions (mesial premotor cortex/rostral cingulate zone, dorsolateral prefrontal cortex), parietal regions, insula, contralateral anterior putamen and midbrain in the internally initiated condition vs. the externally triggered condition. |
| **Krieghoff et al. (2009)** | fMRI | Intentional action selection vs. external action selection & Intentional action timing vs. external action timing | Greater activation in the rostral cingulate zone when subjects freely selected which action to perform vs. external action selection; greater activity in the superior medial frontal gyrus when subjects acted based on their internal timing vs. external action timing. |
| **Rosenberg-Katz et al. (2012)** | fMRI | Externally guided action planning vs. internally guided action planning. | Greater activation in the pre-SMA during internally compared to externally guided action planning. |
| **Hoffstaedter et al. (2013)** | fMRI | Intentional choice vs. no choice condition & Intentional choice vs. timed condition. | Greater activation in the pre-supplementary motor area and dorsal premotor cortex when subjects freely selected which action to perform than the no-choice condition; greater activity in the supplementary motor area, insular cortex, area 44, bilateral anterior putamen, globus pallidus, and left cerebellum subcortically when subjects acted based on their internal timing than the timed condition. |
| **Schel et al. (2014)** | fMRI | Intentional action vs. stimulus-driven action. | Greater activation in the bilateral inferior frontal gyrus, bilateral middle frontal gyrus, bilateral superior frontal gyrus, the pre-supplementary motor cortex/anterior cingulate cortex, and bilateral inferior parietal lobule when subjects performed tasks based on internal rather than external cues. |
|  |  |  |  |
| **Lynn et al. (2016)** | fMRI | Intentional choice action vs. forced action. | Greater activation in the left inferior frontal gyrus, left superior frontal gyrus, pre-supplementary motor cortex/rostral cingulate zone, dorsolateral prefrontal cortex and right inferior parietal lobule when subjects performed tasks based on internal rather than external cues. |
|  |  |  |  |
| **Wisniewski et al. (2016)** | fMRI | Intentional choice vs. cued choice. | Greater activation in the medial prefontal cortex, dorso-lateral and anterior lateral prefrontal cortex and the parietal cortex when subjects performed tasks based on internal than external cues. |
|  |  |  |  |
| **Dall’Acqua et al. (2018)** | fMRI | Intentional choice vs. cued choice. | Greater activation in the bilateral inferior parietal lobule, pre-supplementary motor cortex/anterior cingulate cortex, the left anterior insula, the right premotor cortex and the bilateral dorsolateral prefrontal cortex when subjects performed tasks based on internal than external cues. |
| **Zapparoli et al. (2018)** | fMRI | Intentional choice vs. no choice condition. | Greater activation in the pre-supplementary motor area, the anterior cingulate cortex, the middle frontal gyrus and the cerebellum bilaterally, the right inferior parietal lobule, the left inferior frontal gyrus, the middle temporal gyrus, the insula and the midline at the junction between the pons and the brainstem in the intentionally driven condition rather than stimulus-driven condition. |

*Data analysis*

The analyses were performed using the software Gingerale (version 3.0.2) implementing the ALE algorithm (Activation Likelihood Estimation, Eickhoff et al., 2009). All the Talairach coordinates were converted to MNI space using the TAL to MNI_SPM function implemented in GingerALE. Eighteen activation foci fell outside the conservative mask of the GingerALE software (version 3.0.2) and were excluded. For the meta-analysis, we used the Turkeltaub non-additive method (Turkeltaub et al., 2012), with the cluster-forming statistical threshold of p < 0.05 FWE-corrected, single-voxel threshold of p < 0.001 and 1000 Permutations.

*Results*

The ALE meta-analysis of the 24 studies uncovered a total of 11 clusters. The clusters are broadly distributed among cortical and subcortical areas, with a clear predominance of activations in the medial frontal cortex and parietal lobule. **Fig. 1** shows the activation clusters resulting from the analysis, and **Table S13** shows the details of the obtained results expressed in MNI coordinates.

**Table S13. Results of the ALE analysis of the 24 studies on voluntary action. For each cluster, the cluster number (#CL), the anatomical location and the coordinates in the MNI stereotaxic space of the centroid, the ALE and the Z scores were reported.**

| **CL** | **Brain regions (BA)** | **MNI coordinates** | | | | | | **ALE score** | **Z score** |
| --- | --- | --- | --- | --- | --- | --- | --- | --- | --- |
|  |  | **Right Hemisphere** | | | **Left Hemisphere** | | |  |  |
|  |  | **x** | **y** | **z** | **x** | **y** | **z** |  |  |
| 1 | Superior Frontal Gyrus |  |  |  | 0 | 20 | 42 | 0.059 | 9.323 |
|  | Middle Cingulum (24) | 6 | 28 | 32 |  |  |  | 0.031 | 5.997 |
|  | Pre-supplementary motor area (6) | 4 | 6 | 56 |  |  |  | 0.023 | 4.910 |
|  |  | 10 | 22 | 54 |  |  |  | 0.019 | 4.182 |
|  | Supplementary motor area (6) |  |  |  | -6 | 10 | 52 | 0.014 | 3.368 |
| 2 | Inferior Frontal gyrus pars triangularis (45) | 44 | 34 | 28 |  |  |  | 0.023 | 4.910 |
|  | Middle Frontal gyrus (46) | 34 | 44 | 20 |  |  |  | 0.022 | 4.769 |
|  |  | 46 | 42 | 20 |  |  |  | 0.022 | 4.766 |
|  |  | 40 | 44 | 26 |  |  |  | 0.022 | 4.706 |
| 3 | Inferior Parietal lobule (40) | 44 | -44 | 46 |  |  |  | 0.029 | 5.661 |
|  |  | 50 | -46 | 52 |  |  |  | 0.027 | 5.497 |
|  |  | 40 | -42 | 38 |  |  |  | 0.025 | 5.092 |
|  |  | 32 | -48 | 42 |  |  |  | 0.024 | 4.963 |
|  | Supramarginal gyrus (40) | 52 | -40 | 46 |  |  |  | 0.021 | 4.581 |
| 4 | Middle Frontal gyrus (46) |  |  |  | -40 | 38 | 24 | 0.034 | 6.392 |
|  |  |  |  |  | -40 | 30 | 30 | 0.023 | 4.855 |
| 5 | Inferior Frontal gyrus pars opercularis |  |  |  | -50 | 14 | 2 | 0.027 | 5.384 |
|  | Insula |  |  |  | -40 | 12 | -2 | 0.025 | 5.187 |
|  |  |  |  |  | -34 | 16 | 2 | 0.024 | 4.981 |
| 6 | Superior Parietal lobule (7) | 14 | -68 | 54 |  |  |  | 0.023 | 4.824 |
|  | Precuneus (7) | 6 | -68 | 44 |  |  |  | 0.018 | 4.128 |
| 7 | Superior Frontal Gyrus (8) | 20 | 16 | 56 |  |  |  | 0.018 | 4.120 |
|  |  | 18 | 22 | 54 |  |  |  | 0.018 | 4.118 |
|  | Middle Frontal gyrus (8) | 26 | 10 | 54 |  |  |  | 0.018 | 4.101 |
| 8 | Cerebellum crus |  |  |  | -32 | -58 | -34 | 0.033 | 6.267 |
| 9 | Inferior Parietal lobule (40) |  |  |  | -42 | -42 | 42 | 0.024 | 5.070 |
| 10 | Putamen |  |  |  | -20 | 6 | -4 | 0.026 | 5.339 |
| 11 | Insula | 40 | 12 | -4 |  |  |  | 0.019 | 4.257 |
|  |  | 44 | 14 | -8 |  |  |  | 0.018 | 4.088 |

**References**

Blouin, J. S., Bard, C., & Paillard, J. (2004). Contribution of the cerebellum to self-initiated synchronized movements: a PET study. *Exp Brain Res*, *155*(1), 63-68. <https://doi.org/10.1007/s00221-003-1709-9>

Boecker, H., Jankowski, J., Ditter, P., & Scheef, L. (2008). A role of the basal ganglia and midbrain nuclei for initiation of motor sequences. *Neuroimage*, *39*(3), 1356-1369. <https://doi.org/10.1016/j.neuroimage.2007.09.069>

Cunnington, R., Windischberger, C., Deecke, L., & Moser, E. (2002). The preparation and execution of self-initiated and externally-triggered movement: a study of event-related fMRI. *Neuroimage*, *15*(2), 373-385. <https://doi.org/10.1006/nimg.2001.0976>

Dall'Acqua, T., Begliomini, C., Motta, R., Miotto, D., & Castiello, U. (2018). Effects of intentionality and subliminal information in free-choices to inhibit. *Neuropsychologia*, *109*, 28-38. <https://doi.org/10.1016/j.neuropsychologia.2017.11.035>

Deiber, M. P., Passingham, R. E., Colebatch, J. G., Friston, K. J., Nixon, P. D., & Frackowiak, R. S. (1991). Cortical areas and the selection of movement: a study with positron emission tomography. *Exp Brain Res*, *84*(2), 393-402.

Forstmann, B. U., Brass, M., Koch, I., & von Cramon, D. Y. (2006). Voluntary selection of task sets revealed by functional magnetic resonance imaging. *J Cogn Neurosci*, *18*(3), 388-398. <https://doi.org/10.1162/089892906775990589>

Frith, C. D., Friston, K., Liddle, P. F., & Frackowiak, R. S. (1991). Willed action and the prefrontal cortex in man: a study with PET. *Proc Biol Sci*, *244*(1311), 241-246. <https://doi.org/10.1098/rspb.1991.0077>

Hoffstaedter, F., Grefkes, C., Zilles, K., & Eickhoff, S. B. (2013). The "what" and "when" of self-initiated movements. *Cereb Cortex*, *23*(3), 520-530. <https://doi.org/10.1093/cercor/bhr391>

Hunter, M. D., Farrow, T. F., Papadakis, N. G., Wilkinson, I. D., Woodruff, P. W., & Spence, S. A. (2003). Approaching an ecologically valid functional anatomy of spontaneous "willed" action. *Neuroimage*, *20*(2), 1264-1269. <https://doi.org/10.1016/S1053-8119(03)00374-4>

Hyder, F., Phelps, E. A., Wiggins, C. J., Labar, K. S., Blamire, A. M., & Shulman, R. G. (1997). "Willed action": a functional MRI study of the human prefrontal cortex during a sensorimotor task. *Proc Natl Acad Sci U S A*, *94*(13), 6989-6994.

Jahanshahi, M., Jenkins, I. H., Brown, R. G., Marsden, C. D., Passingham, R. E., & Brooks, D. J. (1995). Self-initiated versus externally triggered movements. I. An investigation using measurement of regional cerebral blood flow with PET and movement-related potentials in normal and Parkinson's disease subjects. *Brain*, *118 ( Pt 4)*, 913-933.

Jenkins, I. H., Jahanshahi, M., Jueptner, M., Passingham, R. E., & Brooks, D. J. (2000). Self-initiated versus externally triggered movements. II. The effect of movement predictability on regional cerebral blood flow. *Brain*, *123 ( Pt 6)*, 1216-1228.

Krieghoff, V., Brass, M., Prinz, W., & Waszak, F. (2009). Dissociating what and when of intentional actions. *Front Hum Neurosci*, *3*, 3. <https://doi.org/10.3389/neuro.09.003.2009>

Lau, H. C., Rogers, R. D., Ramnani, N., & Passingham, R. E. (2004). Willed action and attention to the selection of action. *Neuroimage*, *21*(4), 1407-1415. <https://doi.org/10.1016/j.neuroimage.2003.10.034>

Lynn, M. T., Demanet, J., Krebs, R. M., Van Dessel, P., & Brass, M. (2016). Voluntary inhibition of pain avoidance behavior: an fMRI study. *Brain Struct Funct*, *221*(3), 1309-1320. <https://doi.org/10.1007/s00429-014-0972-9>

Mueller, V. A., Brass, M., Waszak, F., & Prinz, W. (2007). The role of the preSMA and the rostral cingulate zone in internally selected actions. *Neuroimage*, *37*(4), 1354-1361. <https://doi.org/10.1016/j.neuroimage.2007.06.018>

Rosenberg-Katz, K., Jamshy, S., Singer, N., Podlipsky, I., Kipervasser, S., Andelman, F.,…Hendler, T. (2012). Enhanced functional synchronization of medial and lateral PFC underlies internally-guided action planning. *Front Hum Neurosci*, *6*, 79. <https://doi.org/10.3389/fnhum.2012.00079>

Rowe, J. B., Stephan, K. E., Friston, K., Frackowiak, R. S., & Passingham, R. E. (2005). The prefrontal cortex shows context-specific changes in effective connectivity to motor or visual cortex during the selection of action or colour. *Cereb Cortex*, *15*(1), 85-95. <https://doi.org/10.1093/cercor/bhh111>

Schel, M. A., Kühn, S., Brass, M., Haggard, P., Ridderinkhof, K. R., & Crone, E. A. (2014). Neural correlates of intentional and stimulus-driven inhibition: a comparison. *Front Hum Neurosci*, *8*, 27. <https://doi.org/10.3389/fnhum.2014.00027>

Thobois, S., Ballanger, B., Baraduc, P., Le Bars, D., Lavenne, F., Broussolle, E., & Desmurget, M. (2007). Functional anatomy of motor urgency. *Neuroimage*, *37*(1), 243-252. <https://doi.org/10.1016/j.neuroimage.2007.04.049>

van Eimeren, T., Wolbers, T., Münchau, A., Büchel, C., Weiller, C., & Siebner, H. R. (2006). Implementation of visuospatial cues in response selection. *Neuroimage*, *29*(1), 286-294. <https://doi.org/10.1016/j.neuroimage.2005.07.014>

Wiese, H., Stude, P., Nebel, K., Forsting, M., & de Greiff, A. (2005). Prefrontal cortex activity in self-initiated movements is condition-specific, but not movement-related. *Neuroimage*, *28*(3), 691-697. <https://doi.org/10.1016/j.neuroimage.2005.06.044>

Wisniewski, D., Goschke, T., & Haynes, J. D. (2016). Similar coding of freely chosen and externally cued intentions in a fronto-parietal network. *Neuroimage*, *134*, 450-458. <https://doi.org/10.1016/j.neuroimage.2016.04.044>

Zapparoli, L., Seghezzi, S., Scifo, P., Zerbi, A., Banfi, G., Tettamanti, M., & Paulesu, E. (2018). Dissecting the neurofunctional bases of intentional action. *Proc Natl Acad Sci U S A*. <https://doi.org/10.1073/pnas.1718891115>
